# Supplementary figures and images for: Computational investigation of cis-1,4-polyisoprene binding to the latex-clearing protein LcpK30
Source: PLoS One. 2024 May 15;19(5):e0302398. doi: 10.1371/journal.pone.0302398 (PMC11095694; doi:10.1371/journal.pone.0302398)

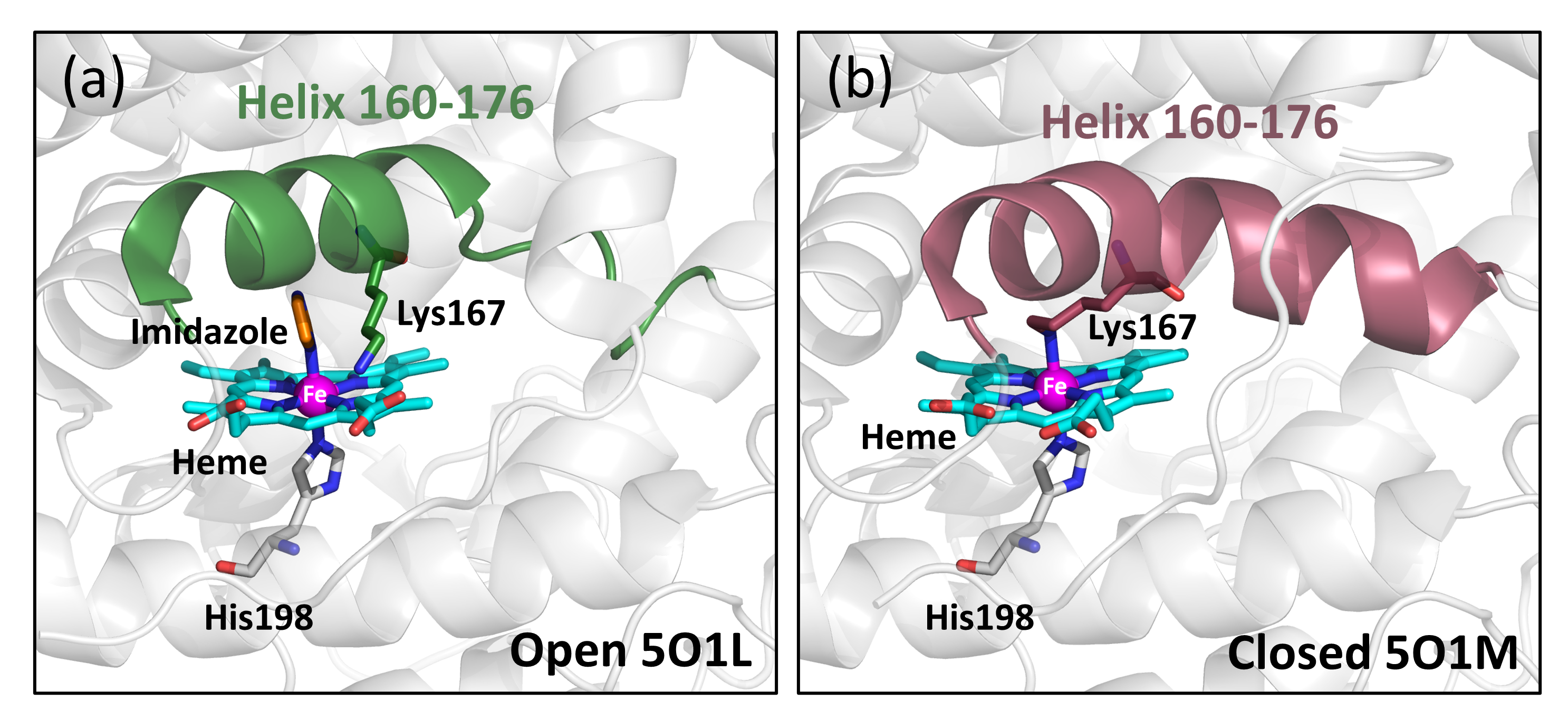

Supplement: S1 Fig — Close view of the active site from the crystal structure of LcpK30 showing the helix 160–176 near heme in (a) open state (PDB: 5O1L) and (b) closed state (PDB: 5O1M). (TIF) [file pone.0302398.s001.tif]

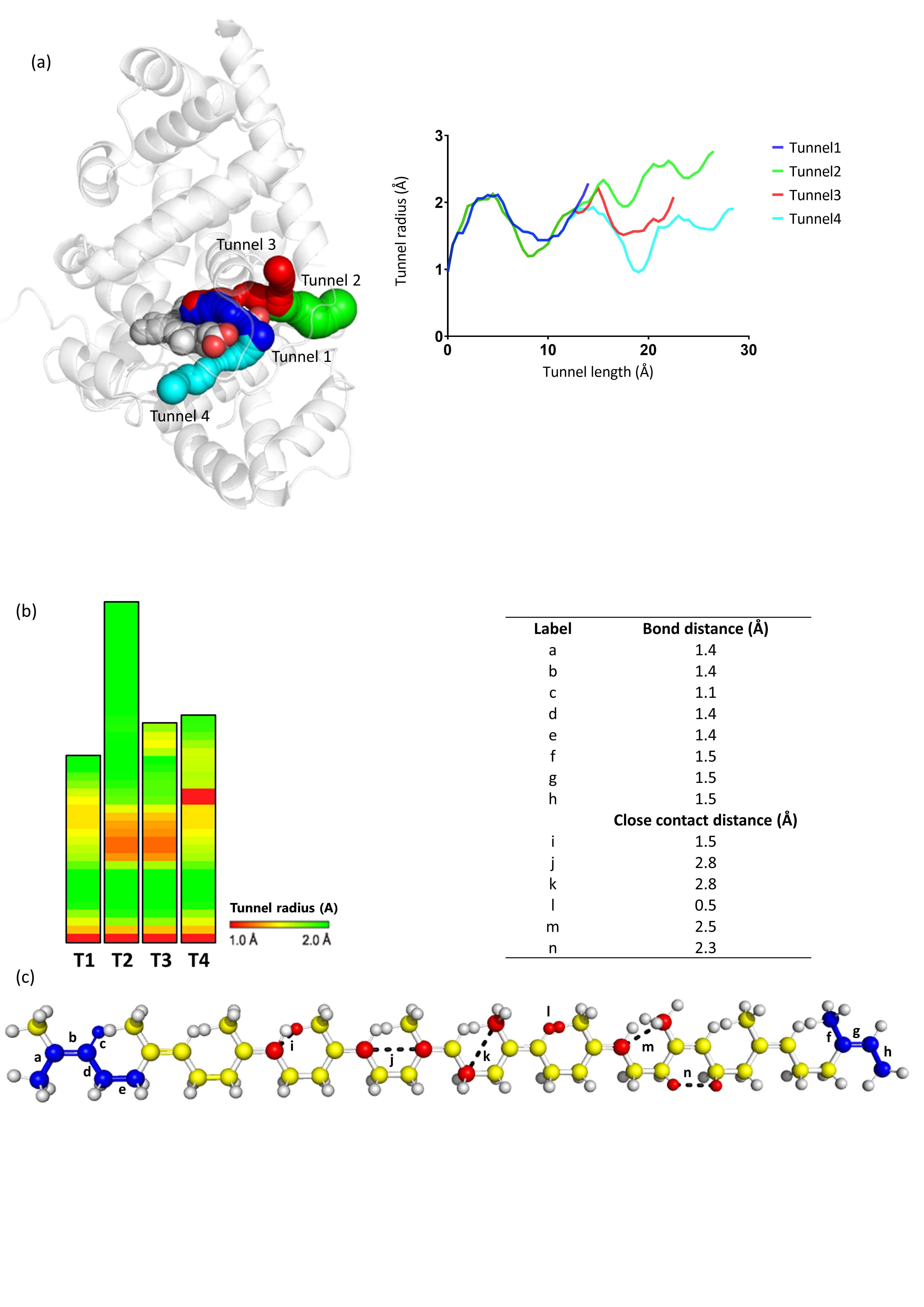

Supplement: S2 Fig — Profiles were calculated using the LcpK30 crystal structure in the open state (PDB 5O1L) using the CAVER-Pymol plugin 3.0.3. (a) Variation of tunnel radius with length; (b) tunnel profile heatmaps; (c) distances between different atoms in the cis-1,4-polyisoprene chains, used to estimate maximum width of the chain in an extended conformation. The distances were measured between the indicated carbon atoms that represent the bond length (blue) and close contact atoms (red). The addition of C-H distances is anticipated to add max. 1.1 Å. (TIF) [file pone.0302398.s002.tif]

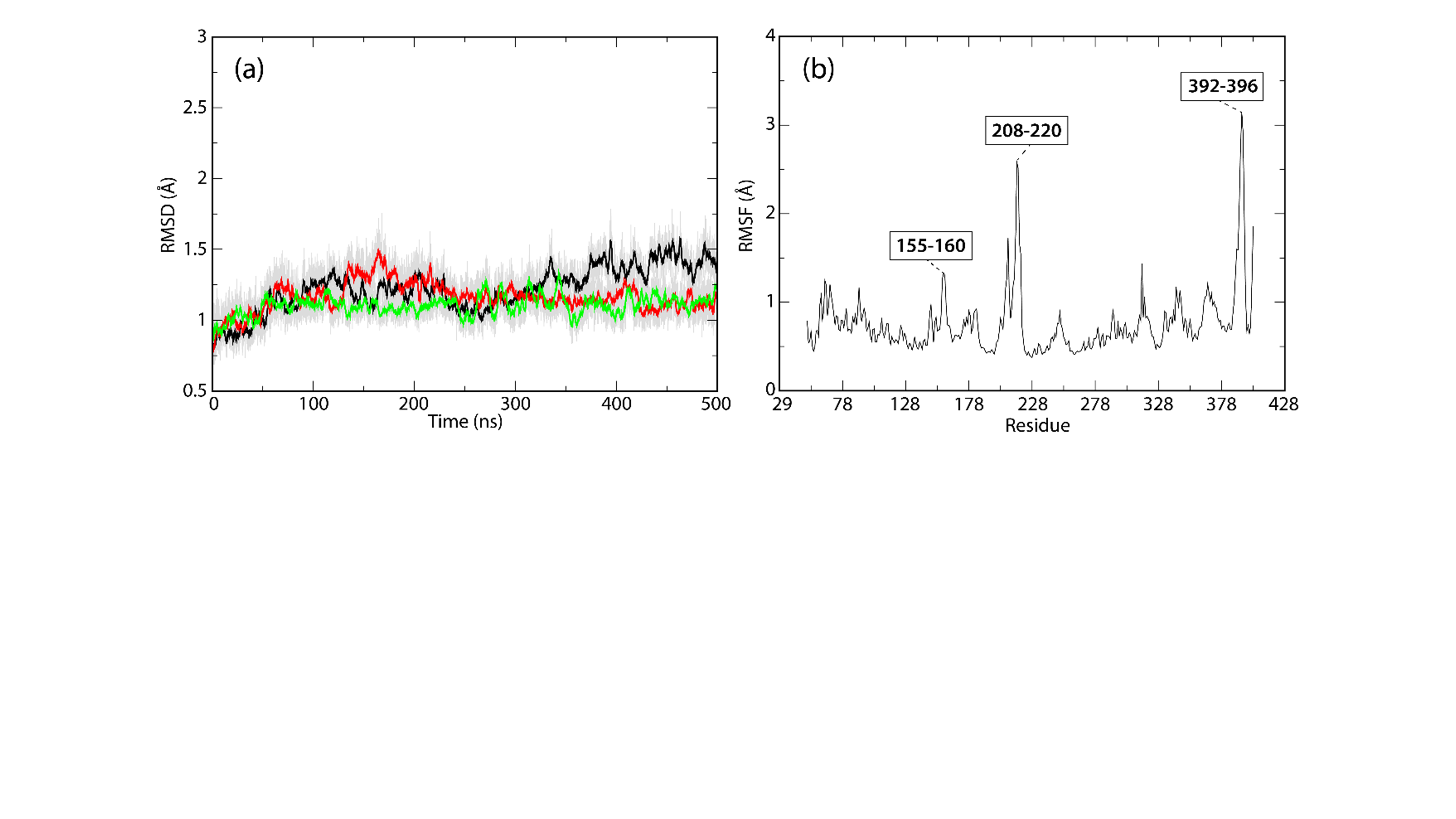

Supplement: S3 Fig — (a) C-alpha RMSD and (b) RMSF values were calculated from 3 independent 500 ns MD simulations (black, red and green) of LcpK30 from the reference X-ray structure in the open state. Due to increased flexibility of the N-terminus, residues 29–49 were omitted from the analysis to minimize the noise. The RMSF values are shown per residue as average from all 3 trajectories. (TIF) [file pone.0302398.s003.tif]

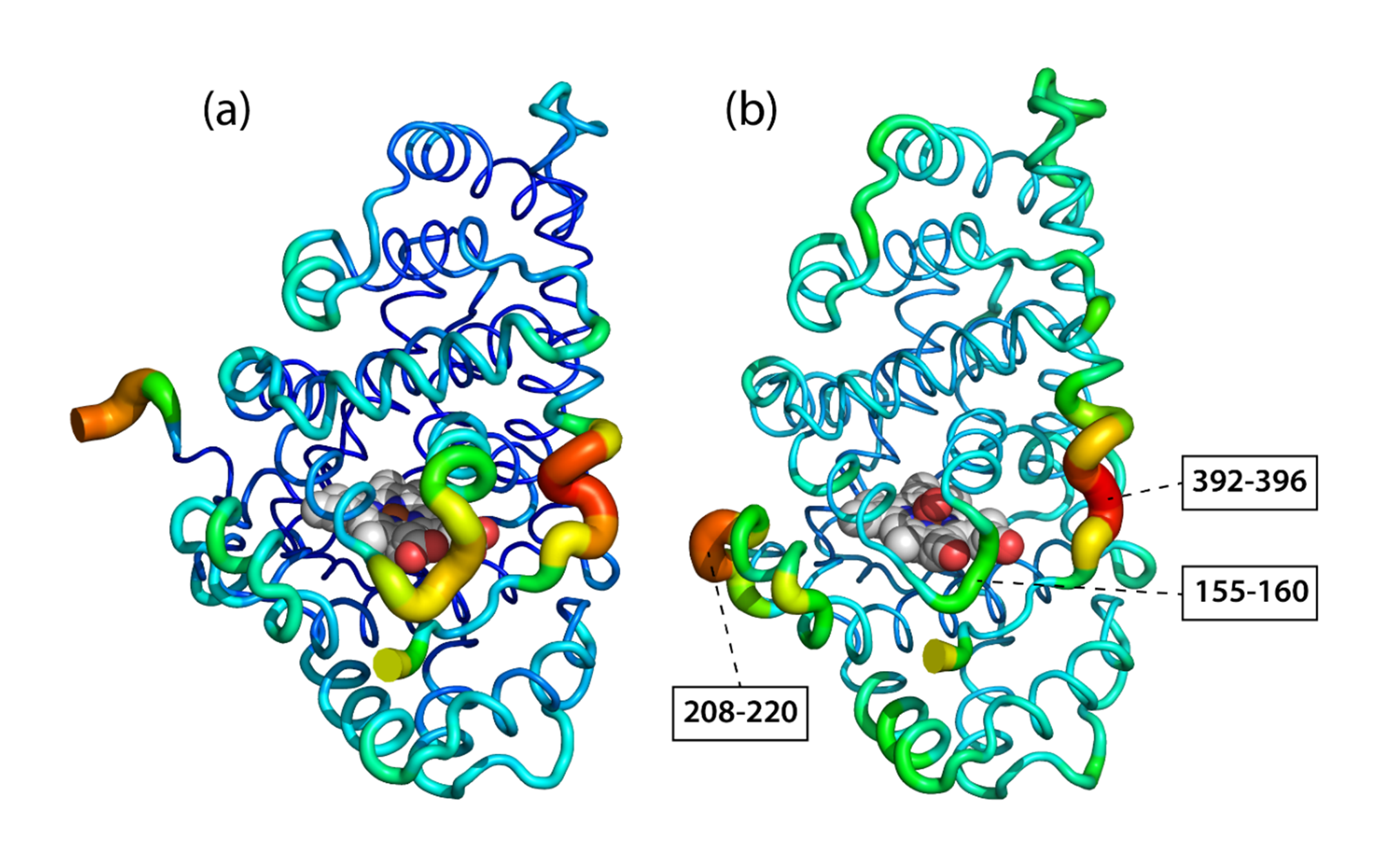

Supplement: S4 Fig — (a) Experimental B-factor from the X-ray structure and (b) C-alpha RMSF (Å) values from MD simulations plotted on the backbone of LcpK30. Due to large fluctuations of the N-terminus, residues 29–49 were omitted from the analysis of the MD trajectories for the sake of clarity. The heme cofactor is represented in spheres (orange = Fe, white = C, red = O, blue = N). The protein structures are depicted with the B-factor putty representation where the backbone is displayed as a tube with a diameter correlated to the experimental B-factor from the X-ray structure (a) or RMSF from MD simulations (b). Thicker tube indicates higher flexibility. The structures are coloured with a continuous scale that ranges from blue to red to indicate the backbone mobility, where blue is low, and red is high backbone flexibility. (TIF) [file pone.0302398.s004.tif]

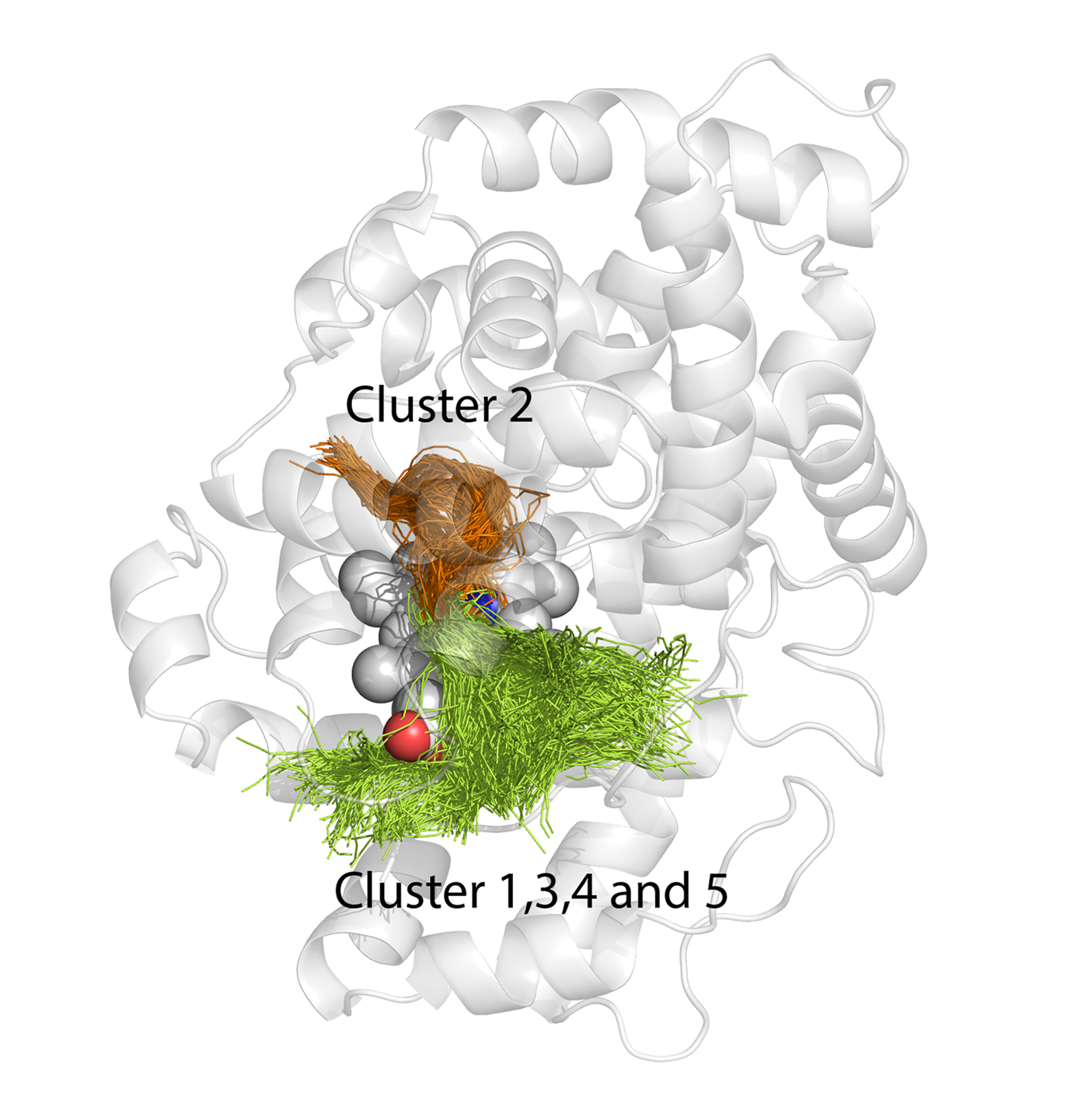

Supplement: S5 Fig — First 5 dominant tunnel clusters were calculated with CAVER Analyst 2 on 1500 snapshots from MD simulations of the closed-like state. Clustering displays the centre lines for all tunnels computed for all snapshots at once. Centre lines are coloured according to their related clusters. Clusters 1, 3, 4 and 5 form one narrow supercluster, colored in limon. (TIF) [file pone.0302398.s005.tif]

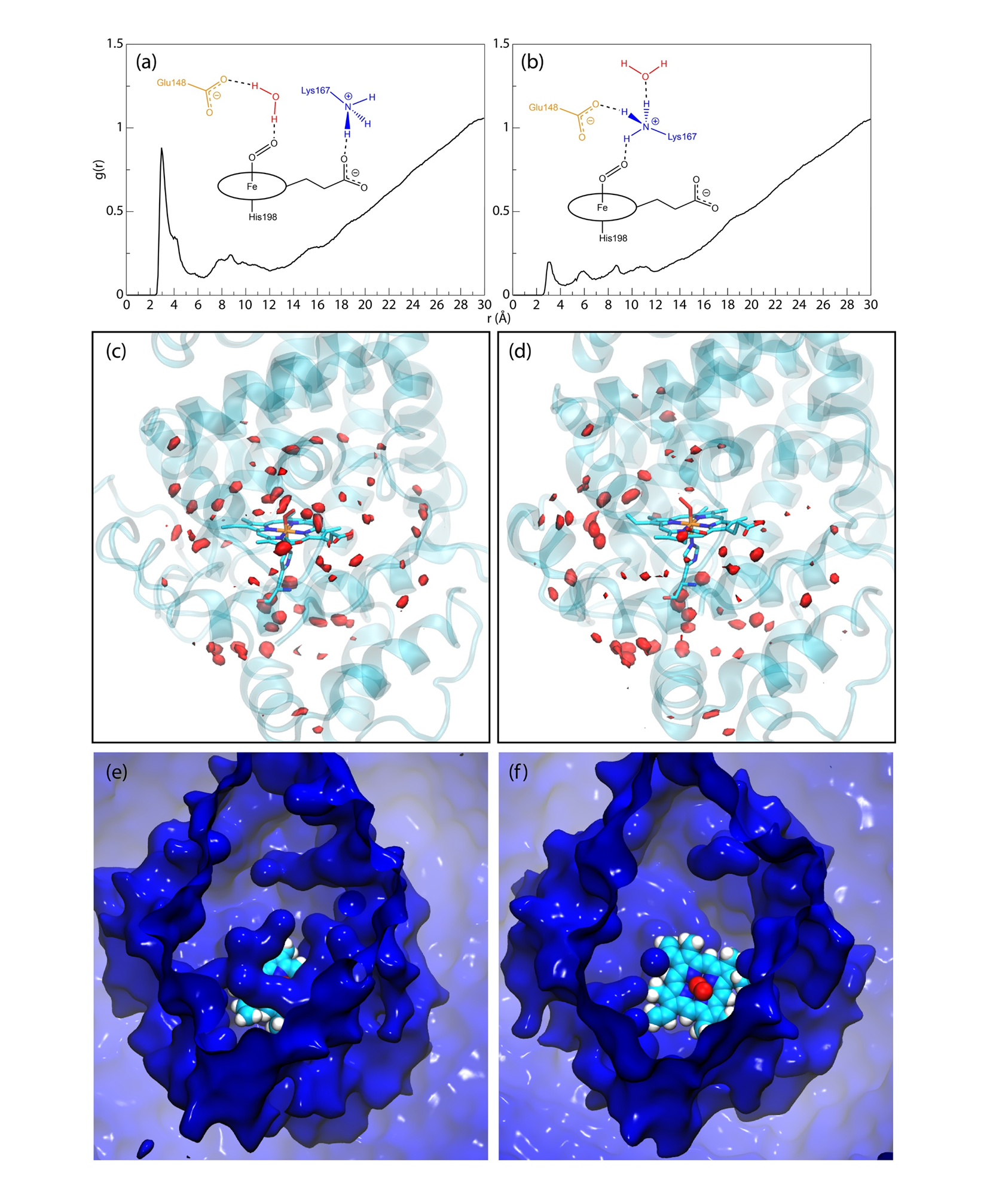

Supplement: S6 Fig — Radial distribution function (RDF) of water oxygen atoms from O2 molecule bound to heme in (a) open and (b) closed LcpK30. The isosurface (isovalue = 100) showing the density of water oxygen atoms around the active site in (c) open and (d) closed LcpK30. Top view at the heme cofactor in the active site of LcpK30 showing water molecules as blue surface using the representative MD snapshots in (e) open and (f) closed LcpK30. The analysis was carried out on 6000 frames from 3 repeat MD simulations of open and closed-like LcpK30 states. (TIF) [file pone.0302398.s006.tif]

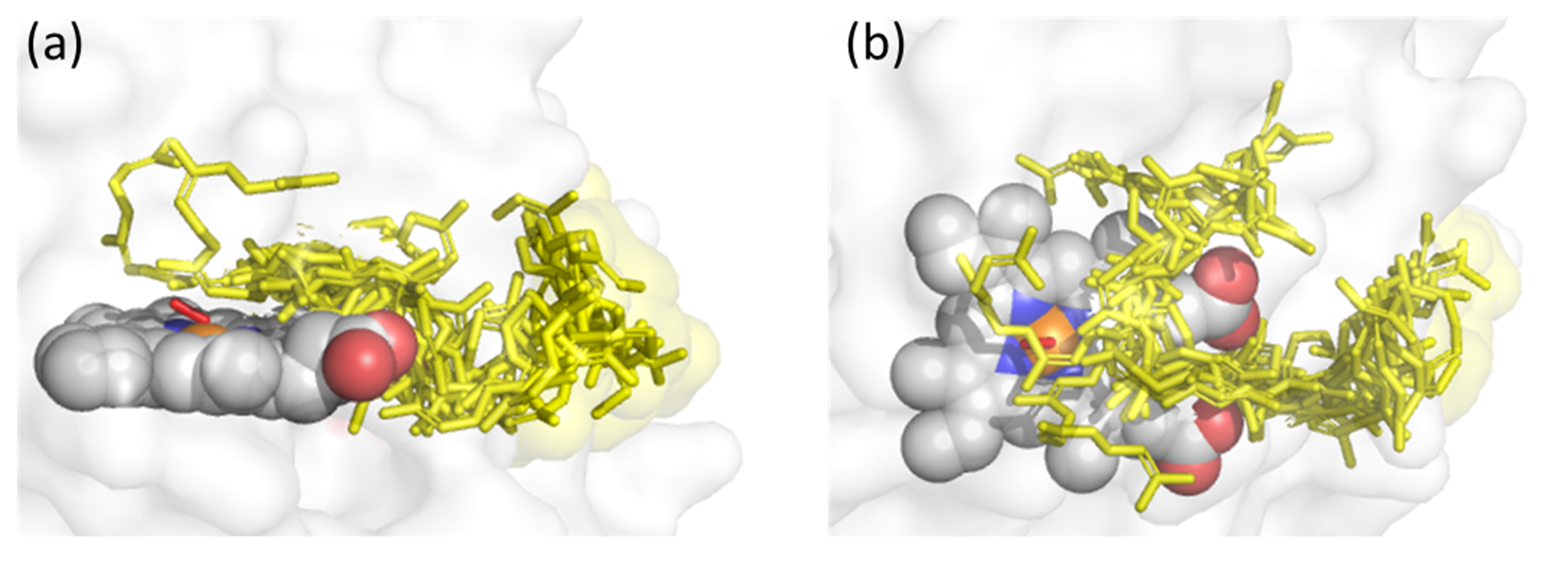

Supplement: S7 Fig — (a) Best 10 poses obtained using the ChemPLP scoring function; (b) best 10 poses obtained using ChemScore scoring function. (TIF) [file pone.0302398.s007.tif]

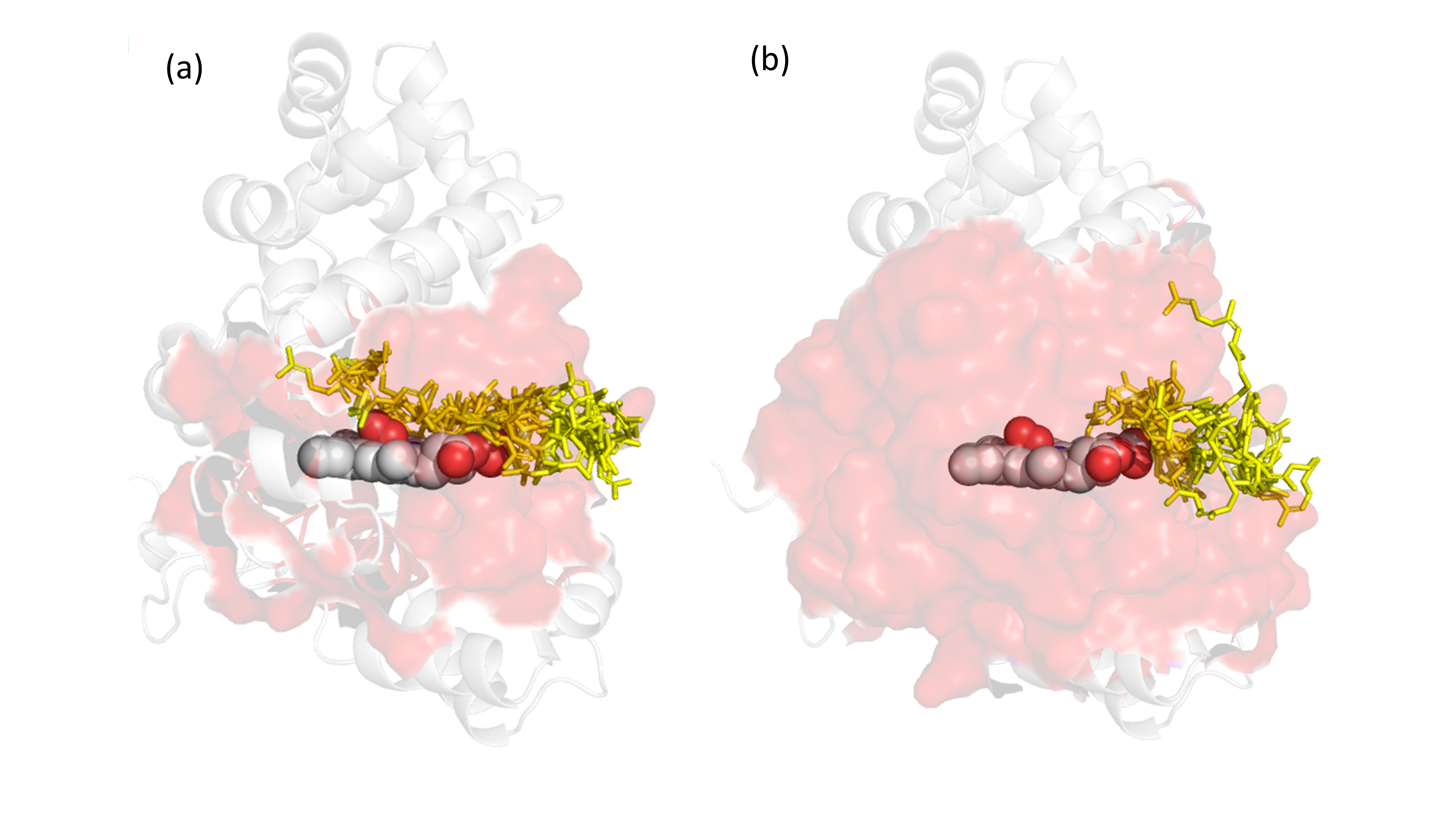

Supplement: S8 Fig — Superimposed docking conformations results of C50H82 within a defined radius of (a) 10 Å and (b) 15 Å from the central iron atom in heme using ChemPLP flexible docking. Ligand is represented as yellow sticks, and the heme cofactor is represented in spheres (white = C, red = O, blue = N). (TIF) [file pone.0302398.s008.tif]

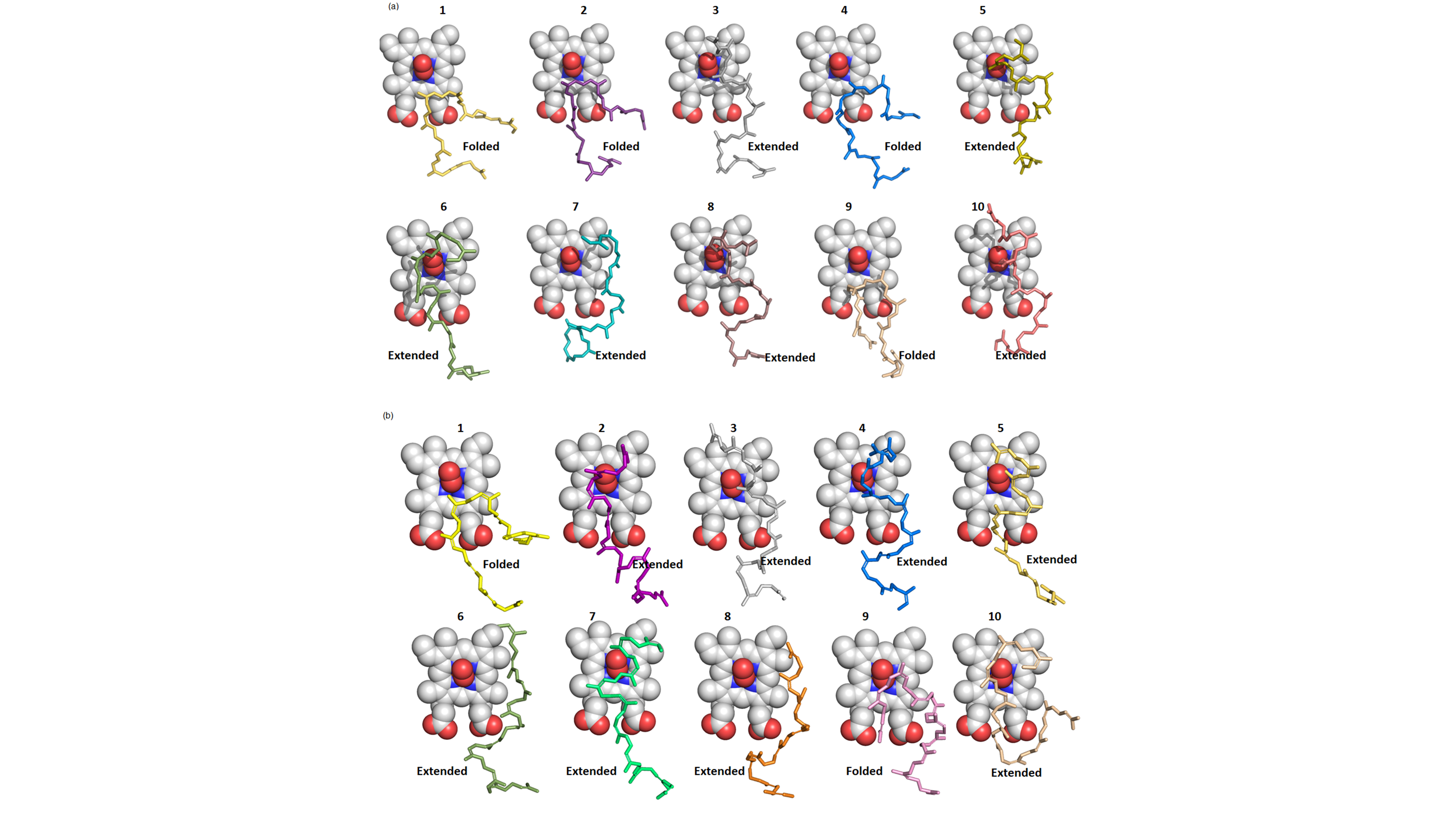

Supplement: S9 Fig — Docking conformations of cis-1,4-polyisoprene (C50H82) near heme. Ranking is by the fitness score from highest to lowest. a) Poses obtained with the ChemPLP fitness function; b) poses obtained with the ChemScore fitness function. Docking solutions were calculated with induced fit docking using a 10 Å binding site. Enzyme and hydrogens were omitted for the sake of clarity. (TIF) [file pone.0302398.s009.tif]

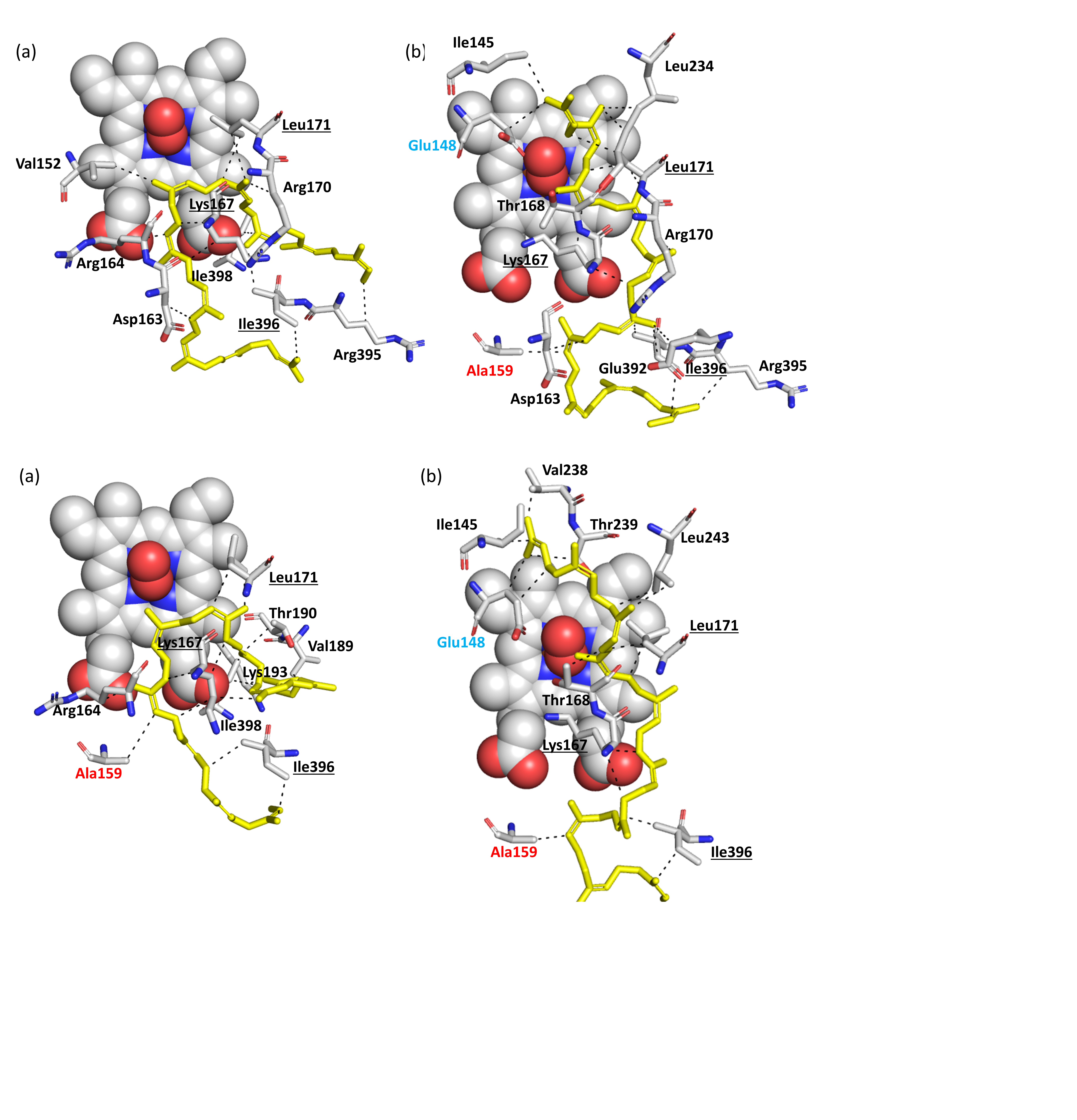

Supplement: S10 Fig — Protein-ligand interactions identified by PLIP analysis. Docked poses were obtained with ChemPLP (above) and ChemScore (below). (a) docking solutions ranked 1, representative of folded conformations and (b) docking solutions ranked 3, representative of extended conformations. Docking solutions were calculated with induced fit docking using a 10 Å binding site and ranked based on the fitness score from highest to lowest (see Table 1). The hydrophobic interactions between the ligand and interacting residues are shown by dashed lines. The three residues that appeared in all interactions are underlined. The residue that interacted only with the extended conformation is coloured in blue. The red coloured residue (Ala159) appeared in all interactions with poses obtained using ChemScore and in most interactions with poses obtained using ChemPLP. (TIF) [file pone.0302398.s010.tif]

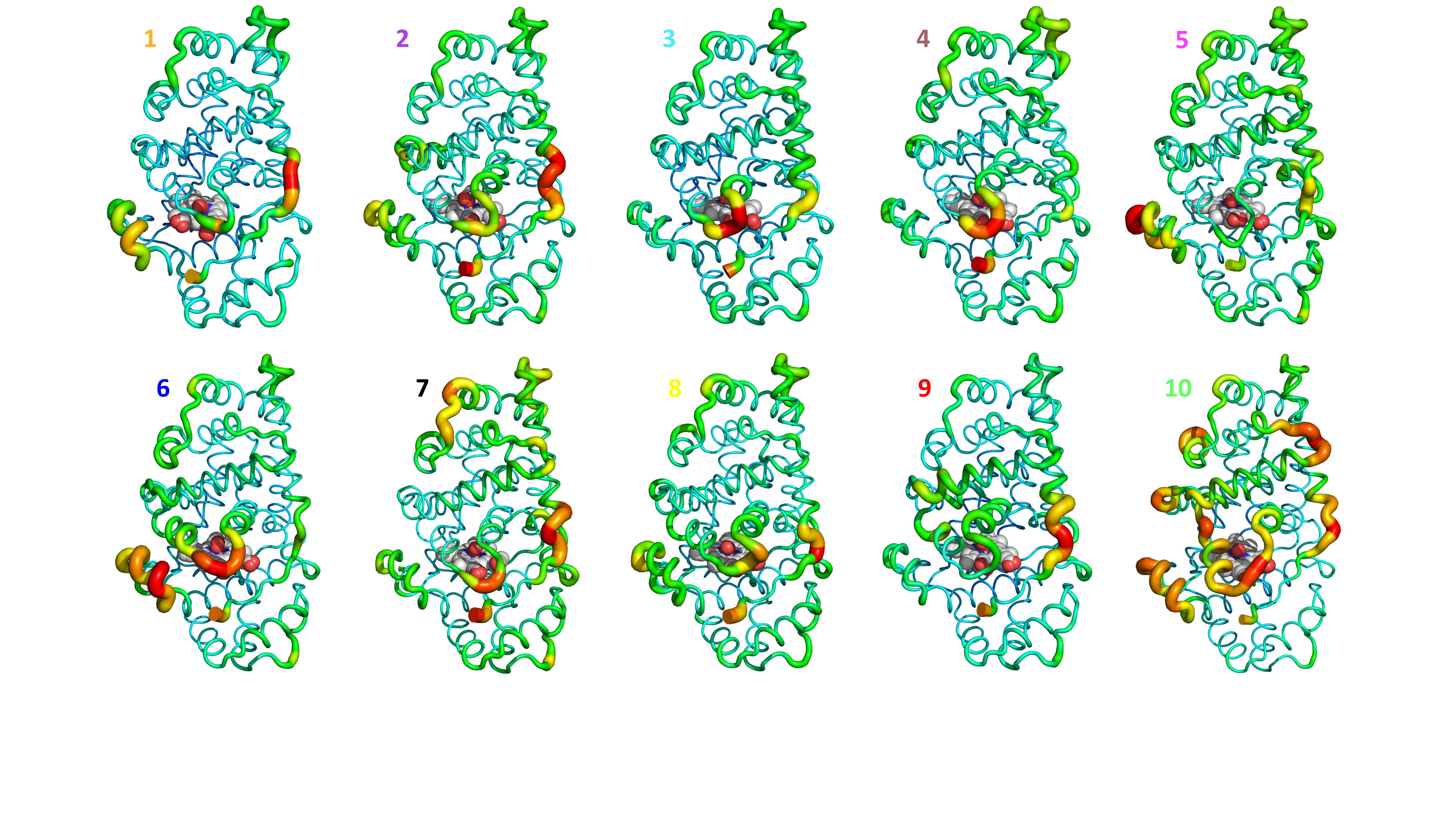

Supplement: S11 Fig — C-alpha RMSF calculated during 100 ns MD simulations of LcpK30 with cis-1,4-polyisoprene bound, starting from 10 docking poses obtained with ChemPLP fitness function. All values are calculated from the reference X-ray structure of the enzyme and projected on the average structure of the enzyme. Due to increased flexibility, residues 29–49 from N-terminus were omitted from the analysis to minimise the noise. The substrate is omitted for the sake of clarity. The protein structures are depicted with the putty representation where the backbone is displayed as a tube with a diameter correlated to the RMSF from MD simulations (thicker tube indicates higher RMSF). The structures are coloured with continuous scale that ranges from blue to red to indicate the backbone mobility, where blue is low and red is high backbone flexibility. (TIF) [file pone.0302398.s011.tif]

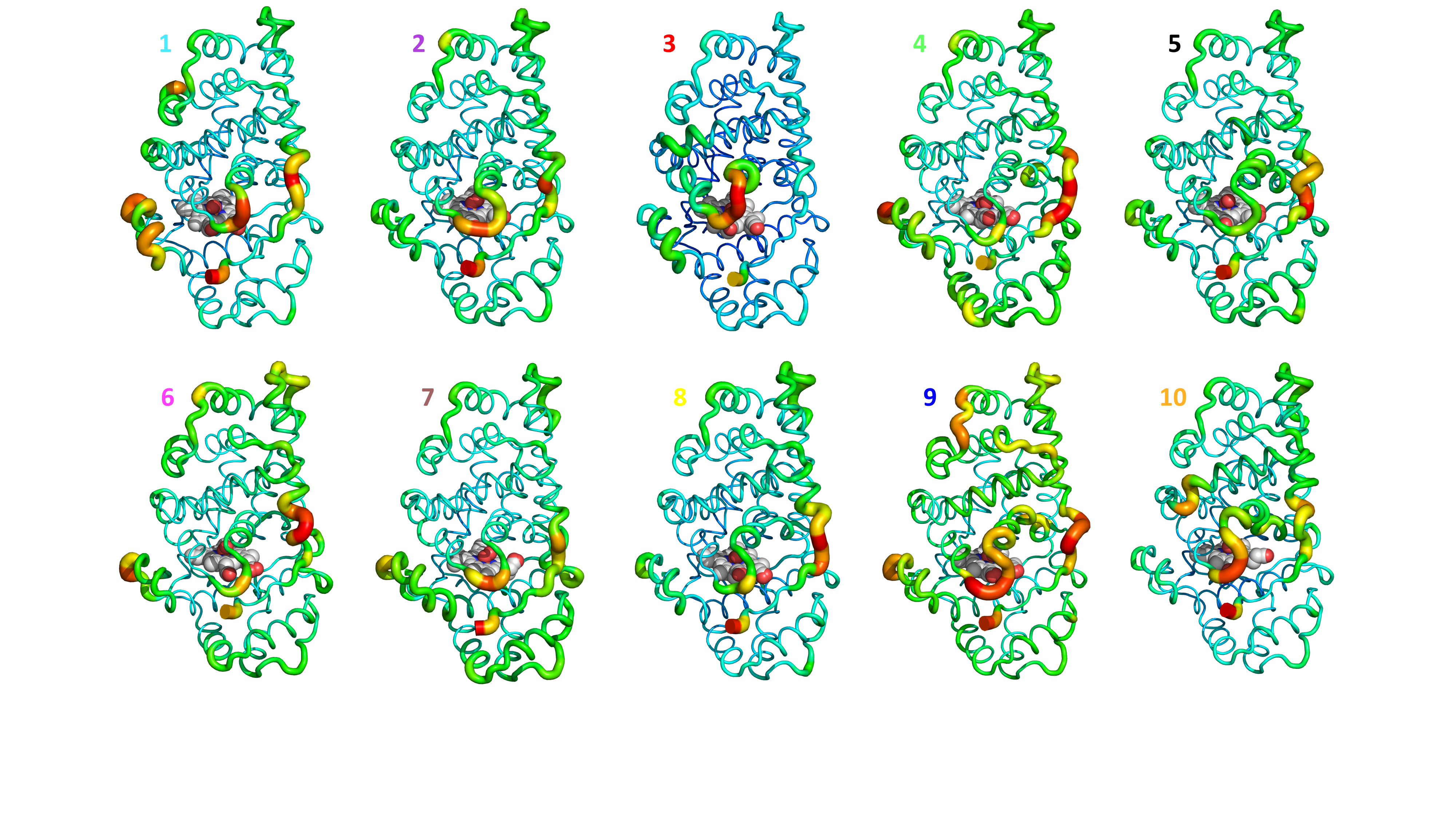

Supplement: S12 Fig — C-alpha RMSF calculated during 100 ns MD simulations of LcpK30 with cis-1,4-polyisoprene bound, starting from 10 docking poses obtained with ChemScore fitness function. All values are calculated from the reference X-ray structure of the enzyme and projected on the average structure of the enzyme. Due to increased flexibility, residues 29–49 from N-terminus were omitted from the analysis to minimise the noise. The substrate is omitted for the sake of clarity. The protein structures are depicted with the putty representation where the backbone is displayed as a tube with a diameter correlated to the RMSF from MD simulations (thicker tube indicates higher RMSF). The structures are coloured with continuous scale that ranges from blue to red to indicate the backbone mobility, where blue is low and red is high backbone flexibility. (TIF) [file pone.0302398.s012.tif]

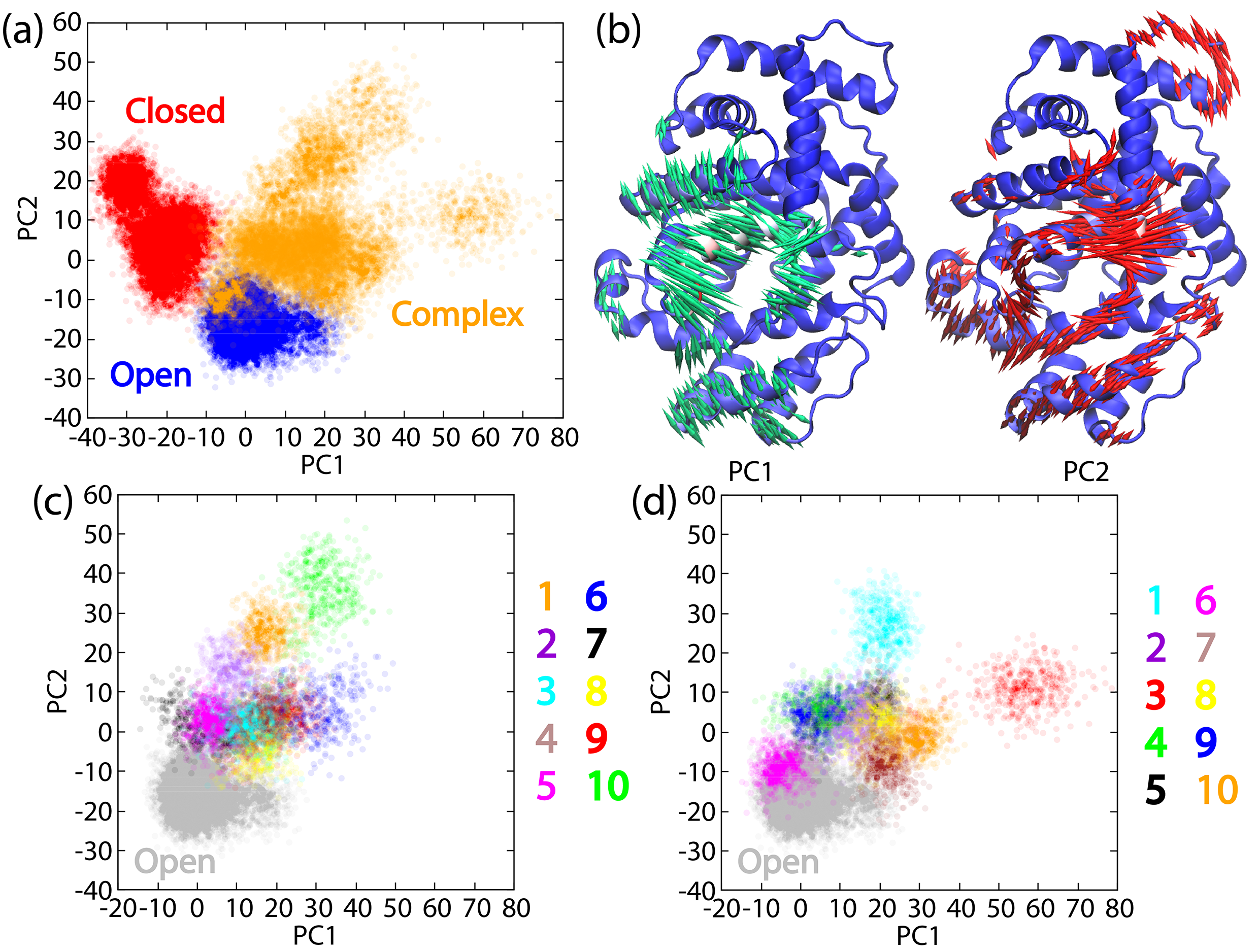

Supplement: S13 Fig — PCA was performed considering cartesian coordinates of protein backbone atoms (N, Cα, C and O). (a) MD snapshots projected on the first two principal components. Blue and red circles represent MD snapshots belonging to open and closed-like states, respectively, orange circles represent MD snapshots belonging to the open state LcpK£0 in complex with the substrate. (b) Normal mode displacement vectors associated with the first two principal components showing only motions longer than 2 Å as porcupine in both directions. The backbone is coloured by the mobility where lower and higher flexibility is depicted in blue and red, respectively. Structures from MD simulations projected on the first two principal components considering open LcpK30 (grey) and LcpK30 in complex with substrate from (c) ChemPLP and (d) ChemScore docking poses. (TIF) [file pone.0302398.s013.tif]

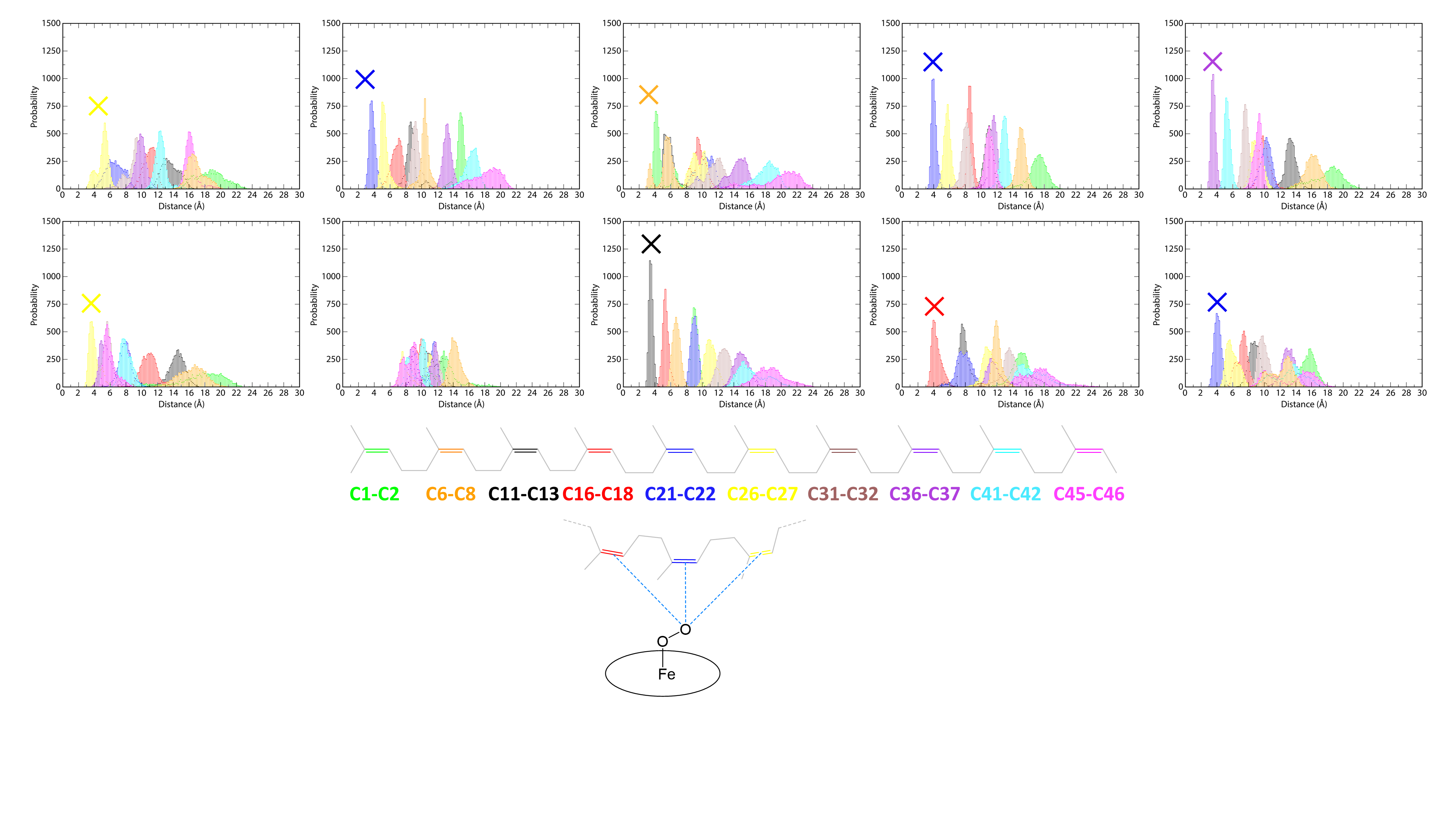

Supplement: S14 Fig — Calculations were during 100 ns MD simulations of LcpK30 with cis-1,4-polyisoprene bound, starting from 10 docking poses obtained with ChemPLP fitness score. The cleaved bonds were the ones found closest to O2. The color of the C = C double bond (see scheme at the bottom) corresponds to the color of the probability histogram. (TIF) [file pone.0302398.s014.tif]

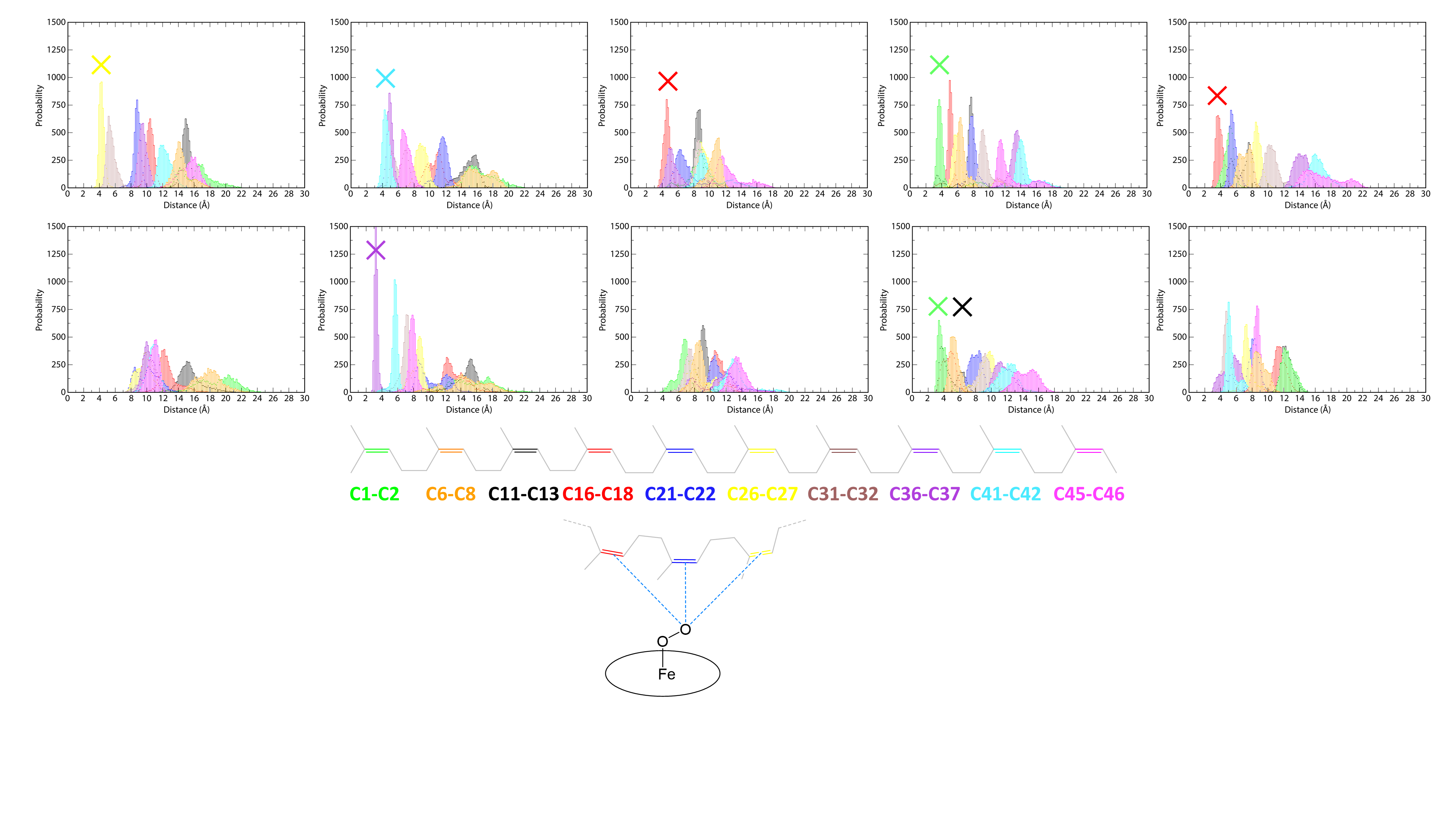

Supplement: S15 Fig — Calculations were during 100 ns MD simulations of LcpK30 with cis-1,4-polyisoprene bound, starting from 10 docking poses obtained with ChemScore fitness score. The cleaved bonds were the ones found closest to O2. The color of the C = C double bond (see scheme at the bottom) corresponds to the color of the probability histogram. (TIF) [file pone.0302398.s015.tif]
