# Supplementary material for: Computational investigation of cis-1,4-polyisoprene binding to the latex-clearing protein LcpK30
Source: PLoS One. 2024 May 15;19(5):e0302398. doi: 10.1371/journal.pone.0302398 (PMC11095694; doi:10.1371/journal.pone.0302398)
Supplement: S1 Table — (PPTX) [file pone.0302398.s016.pptx]

## Slide 1
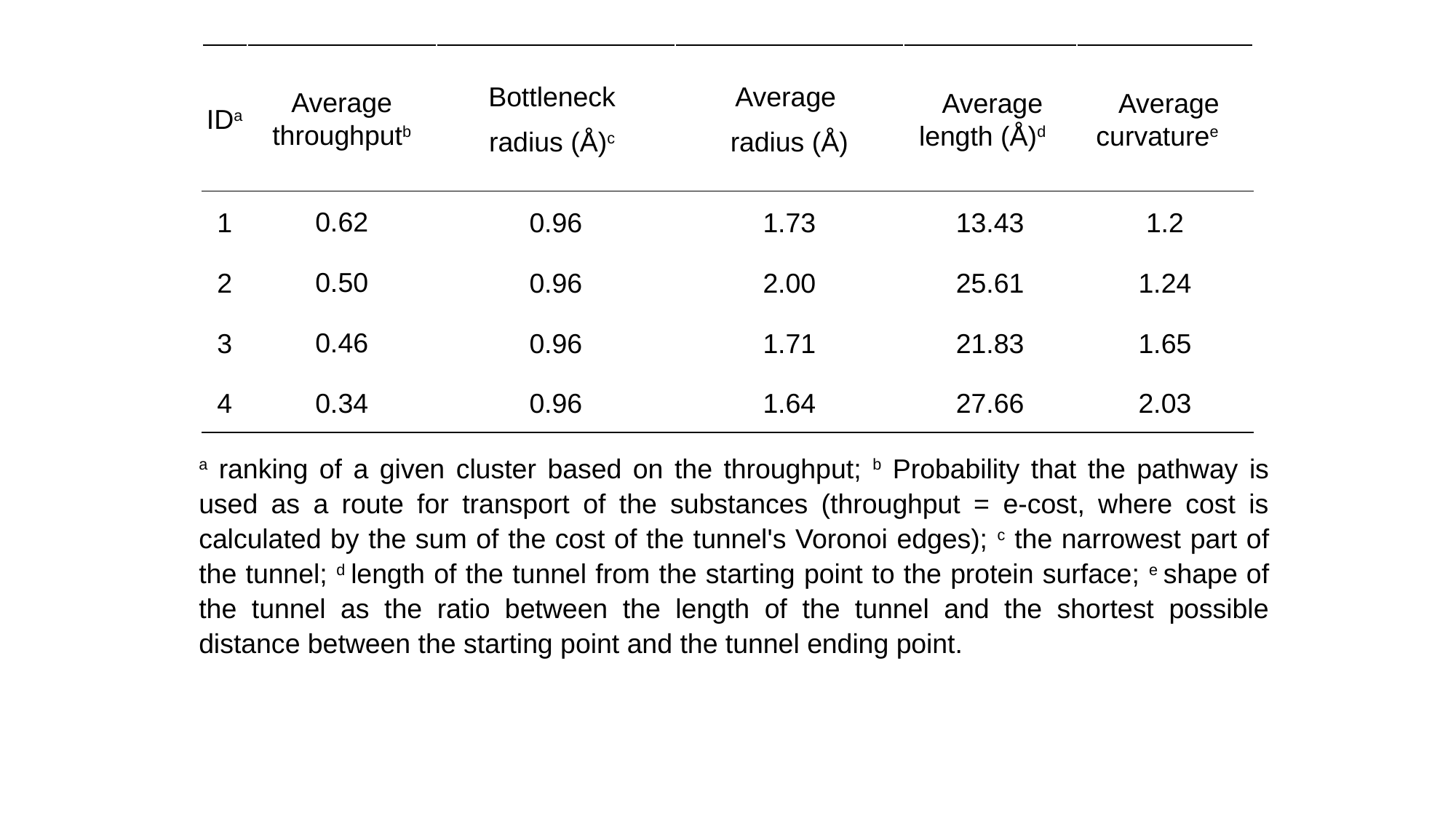

| IDa | Average throughputb | Bottleneck radius (Å)c | Average radius (Å) | Average length (Å)d | Average curvaturee |
| --- | --- | --- | --- | --- | --- |
| 1 | 0.62 | 0.96 | 1.73 | 13.43 | 1.2 |
| 2 | 0.50 | 0.96 | 2.00 | 25.61 | 1.24 |
| 3 | 0.46 | 0.96 | 1.71 | 21.83 | 1.65 |
| 4 | 0.34 | 0.96 | 1.64 | 27.66 | 2.03 |
a ranking of a given cluster based on the throughput; b Probability that the pathway is used as a route for transport of the substances (throughput = e-cost, where cost is calculated by the sum of the cost of the tunnel's Voronoi edges); c the narrowest part of the tunnel; d length of the tunnel from the starting point to the protein surface; e shape of the tunnel as the ratio between the length of the tunnel and the shortest possible distance between the starting point and the tunnel ending point.
