# Supplementary material for: Computational investigation of cis-1,4-polyisoprene binding to the latex-clearing protein LcpK30
Source: PLoS One. 2024 May 15;19(5):e0302398. doi: 10.1371/journal.pone.0302398 (PMC11095694; doi:10.1371/journal.pone.0302398)
Supplement: S2 Table — Solutions are ranked based on the fitness score from highest to lowest. (PPTX) [file pone.0302398.s017.pptx]

## Slide 1
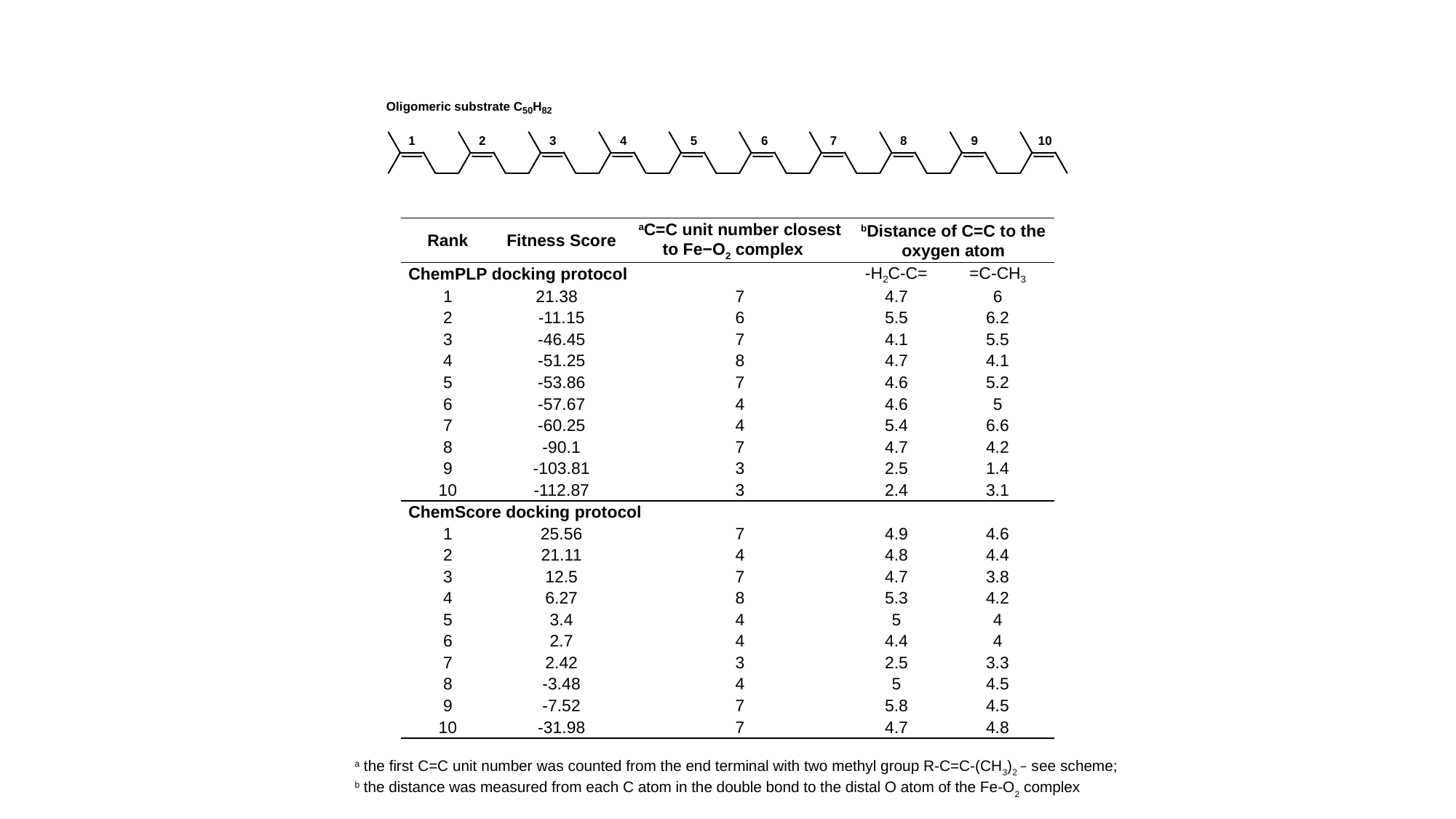

| Rank | Fitness Score | aC=C unit number closest to Fe−O2 complex | bDistance of C=C to the oxygen atom | |
| --- | --- | --- | --- | --- |
| ChemPLP docking protocol | | | -H2C-C= | =C-CH3 |
| 1 | 21.38 | 7 | 4.7 | 6 |
| 2 | -11.15 | 6 | 5.5 | 6.2 |
| 3 | -46.45 | 7 | 4.1 | 5.5 |
| 4 | -51.25 | 8 | 4.7 | 4.1 |
| 5 | -53.86 | 7 | 4.6 | 5.2 |
| 6 | -57.67 | 4 | 4.6 | 5 |
| 7 | -60.25 | 4 | 5.4 | 6.6 |
| 8 | -90.1 | 7 | 4.7 | 4.2 |
| 9 | -103.81 | 3 | 2.5 | 1.4 |
| 10 | -112.87 | 3 | 2.4 | 3.1 |
| ChemScore docking protocol | | | | |
| 1 | 25.56 | 7 | 4.9 | 4.6 |
| 2 | 21.11 | 4 | 4.8 | 4.4 |
| 3 | 12.5 | 7 | 4.7 | 3.8 |
| 4 | 6.27 | 8 | 5.3 | 4.2 |
| 5 | 3.4 | 4 | 5 | 4 |
| 6 | 2.7 | 4 | 4.4 | 4 |
| 7 | 2.42 | 3 | 2.5 | 3.3 |
| 8 | -3.48 | 4 | 5 | 4.5 |
| 9 | -7.52 | 7 | 5.8 | 4.5 |
| 10 | -31.98 | 7 | 4.7 | 4.8 |
a the first C=C unit number was counted from the end terminal with two methyl group R-C=C-(CH3)2 – see scheme; b the distance was measured from each C atom in the double bond to the distal O atom of the Fe-O2 complex
