# Supplementary material for: Computational investigation of cis-1,4-polyisoprene binding to the latex-clearing protein LcpK30
Source: PLoS One. 2024 May 15;19(5):e0302398. doi: 10.1371/journal.pone.0302398 (PMC11095694; doi:10.1371/journal.pone.0302398)
Supplement: S3 Table — Docking solutions are obtained from flexible docking using the ChemPLP scoring function within the GOLD software. The docking was performed in a binding site defined as all atoms within 10 or 15 Å from the central iron atom in heme. The 10 docking solutions are ranked based on the fitness score from highest to lowest. (PPTX) [file pone.0302398.s018.pptx]

## Slide 1
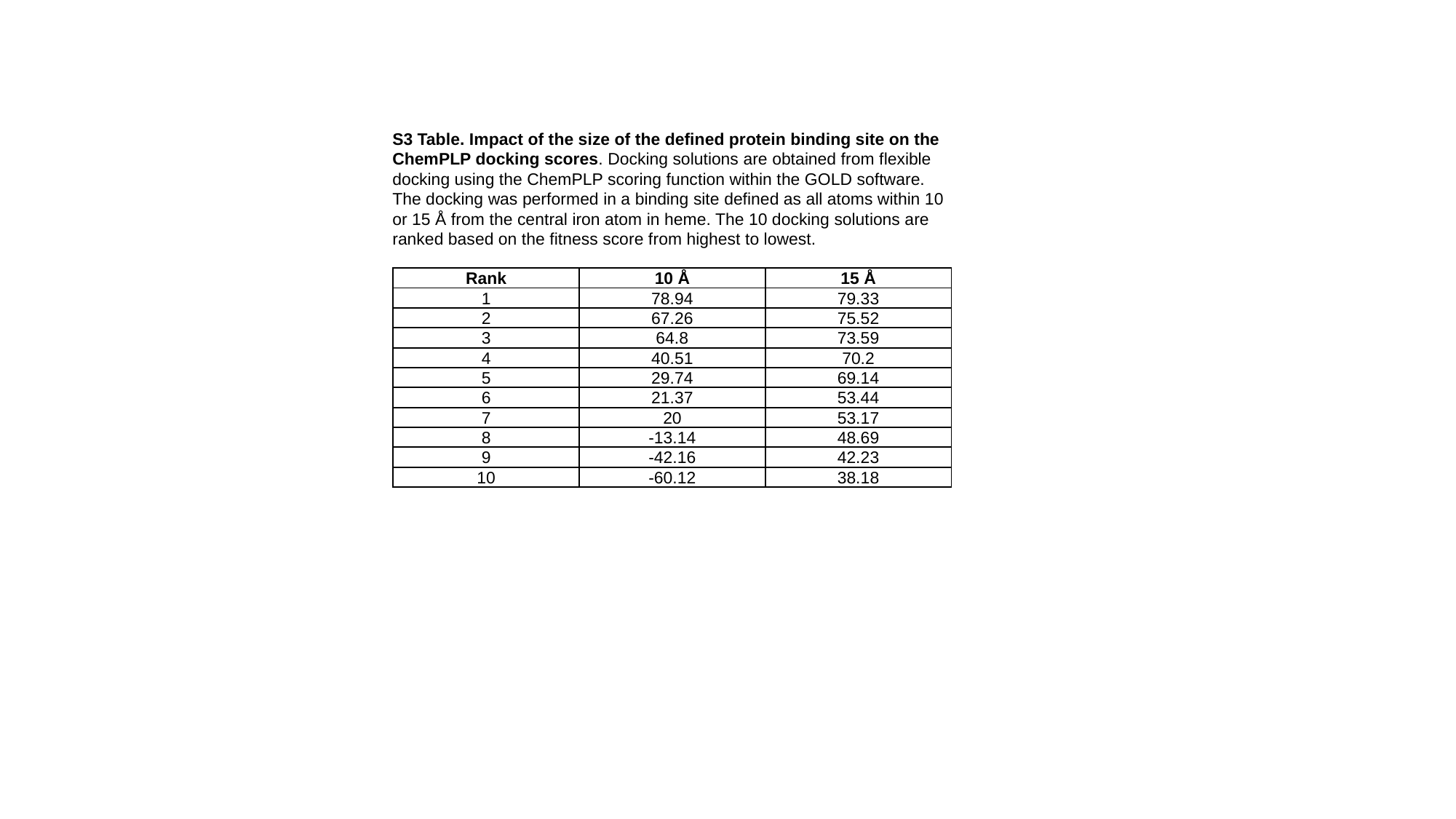

S3 Table. Impact of the size of the defined protein binding site on the ChemPLP docking scores. Docking solutions are obtained from flexible docking using the ChemPLP scoring function within the GOLD software. The docking was performed in a binding site defined as all atoms within 10 or 15 Å from the central iron atom in heme. The 10 docking solutions are ranked based on the fitness score from highest to lowest.
| Rank | 10 Å | 15 Å |
| --- | --- | --- |
| 1 | 78.94 | 79.33 |
| 2 | 67.26 | 75.52 |
| 3 | 64.8 | 73.59 |
| 4 | 40.51 | 70.2 |
| 5 | 29.74 | 69.14 |
| 6 | 21.37 | 53.44 |
| 7 | 20 | 53.17 |
| 8 | -13.14 | 48.69 |
| 9 | -42.16 | 42.23 |
| 10 | -60.12 | 38.18 |
