# Supplementary material for: Computational investigation of cis-1,4-polyisoprene binding to the latex-clearing protein LcpK30
Source: PLoS One. 2024 May 15;19(5):e0302398. doi: 10.1371/journal.pone.0302398 (PMC11095694; doi:10.1371/journal.pone.0302398)
Supplement: S5 Table — Docking solutions were ranked based on the fitness score from highest to lowest. Residues that interact with all / most poses are highlighted in grey and light grey, respectively. (PPTX) [file pone.0302398.s020.pptx]

## Slide 1
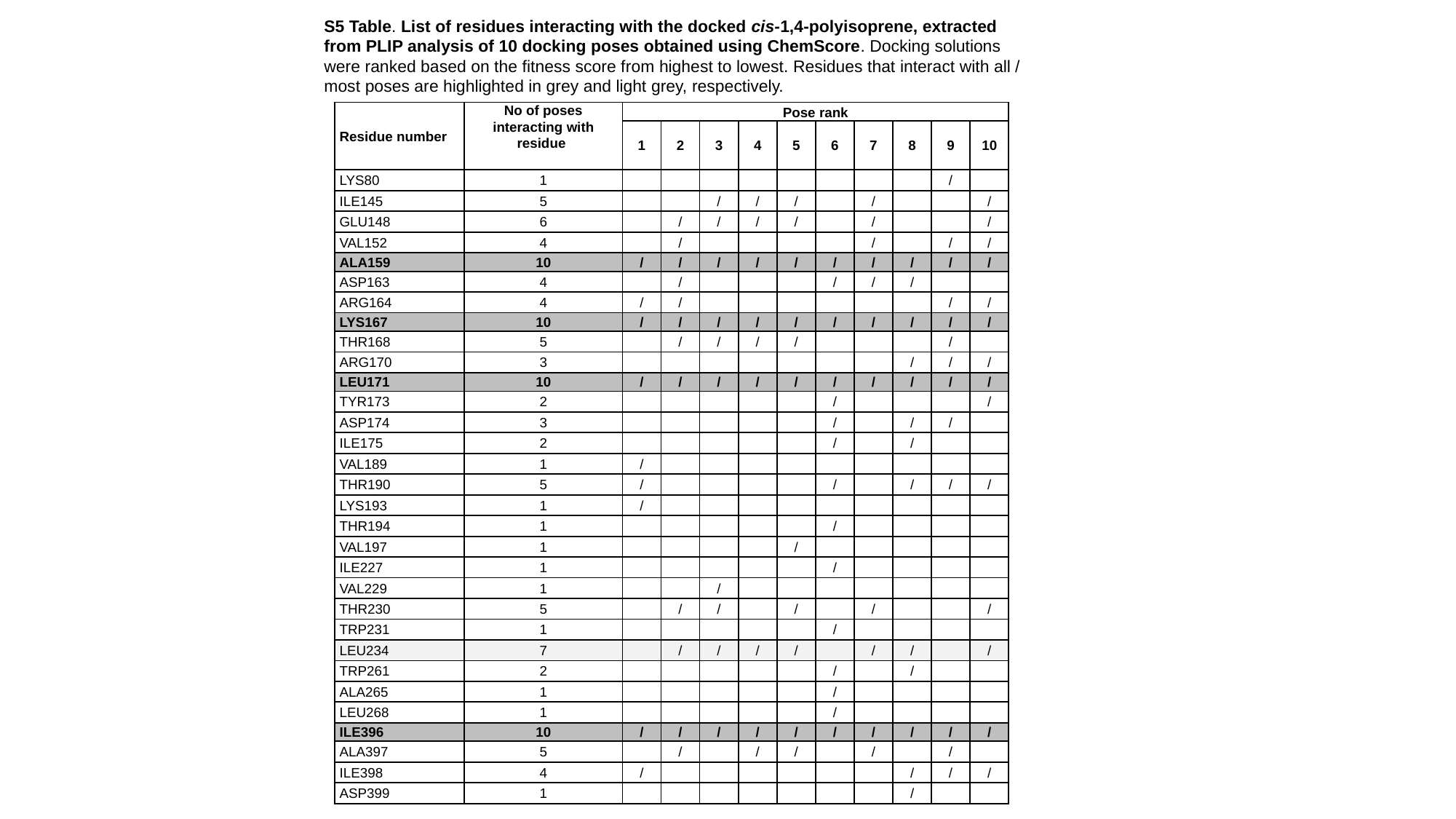

S5 Table. List of residues interacting with the docked cis-1,4-polyisoprene, extracted from PLIP analysis of 10 docking poses obtained using ChemScore. Docking solutions were ranked based on the fitness score from highest to lowest. Residues that interact with all / most poses are highlighted in grey and light grey, respectively.
| Residue number | No of poses interacting with residue | Pose rank | | | | | | | | | |
| --- | --- | --- | --- | --- | --- | --- | --- | --- | --- | --- | --- |
| | | 1 | 2 | 3 | 4 | 5 | 6 | 7 | 8 | 9 | 10 |
| LYS80 | 1 | | | | | | | | | / | |
| ILE145 | 5 | | | / | / | / | | / | | | / |
| GLU148 | 6 | | / | / | / | / | | / | | | / |
| VAL152 | 4 | | / | | | | | / | | / | / |
| ALA159 | 10 | / | / | / | / | / | / | / | / | / | / |
| ASP163 | 4 | | / | | | | / | / | / | | |
| ARG164 | 4 | / | / | | | | | | | / | / |
| LYS167 | 10 | / | / | / | / | / | / | / | / | / | / |
| THR168 | 5 | | / | / | / | / | | | | / | |
| ARG170 | 3 | | | | | | | | / | / | / |
| LEU171 | 10 | / | / | / | / | / | / | / | / | / | / |
| TYR173 | 2 | | | | | | / | | | | / |
| ASP174 | 3 | | | | | | / | | / | / | |
| ILE175 | 2 | | | | | | / | | / | | |
| VAL189 | 1 | / | | | | | | | | | |
| THR190 | 5 | / | | | | | / | | / | / | / |
| LYS193 | 1 | / | | | | | | | | | |
| THR194 | 1 | | | | | | / | | | | |
| VAL197 | 1 | | | | | / | | | | | |
| ILE227 | 1 | | | | | | / | | | | |
| VAL229 | 1 | | | / | | | | | | | |
| THR230 | 5 | | / | / | | / | | / | | | / |
| TRP231 | 1 | | | | | | / | | | | |
| LEU234 | 7 | | / | / | / | / | | / | / | | / |
| TRP261 | 2 | | | | | | / | | / | | |
| ALA265 | 1 | | | | | | / | | | | |
| LEU268 | 1 | | | | | | / | | | | |
| ILE396 | 10 | / | / | / | / | / | / | / | / | / | / |
| ALA397 | 5 | | / | | / | / | | / | | / | |
| ILE398 | 4 | / | | | | | | | / | / | / |
| ASP399 | 1 | | | | | | | | / | | |
