# Supplementary material for: Computational investigation of cis-1,4-polyisoprene binding to the latex-clearing protein LcpK30
Source: PLoS One. 2024 May 15;19(5):e0302398. doi: 10.1371/journal.pone.0302398 (PMC11095694; doi:10.1371/journal.pone.0302398)
Supplement: S6 Table — Docking solutions were initially ranked based on the fitness score from highest to lowest. A cut-off of 5 Å was used and only contacts with total fraction more than 4.5% are listed. Ext = extended conformation; Fold = folded conformation. (PPTX) [file pone.0302398.s021.pptx]

## Slide 1
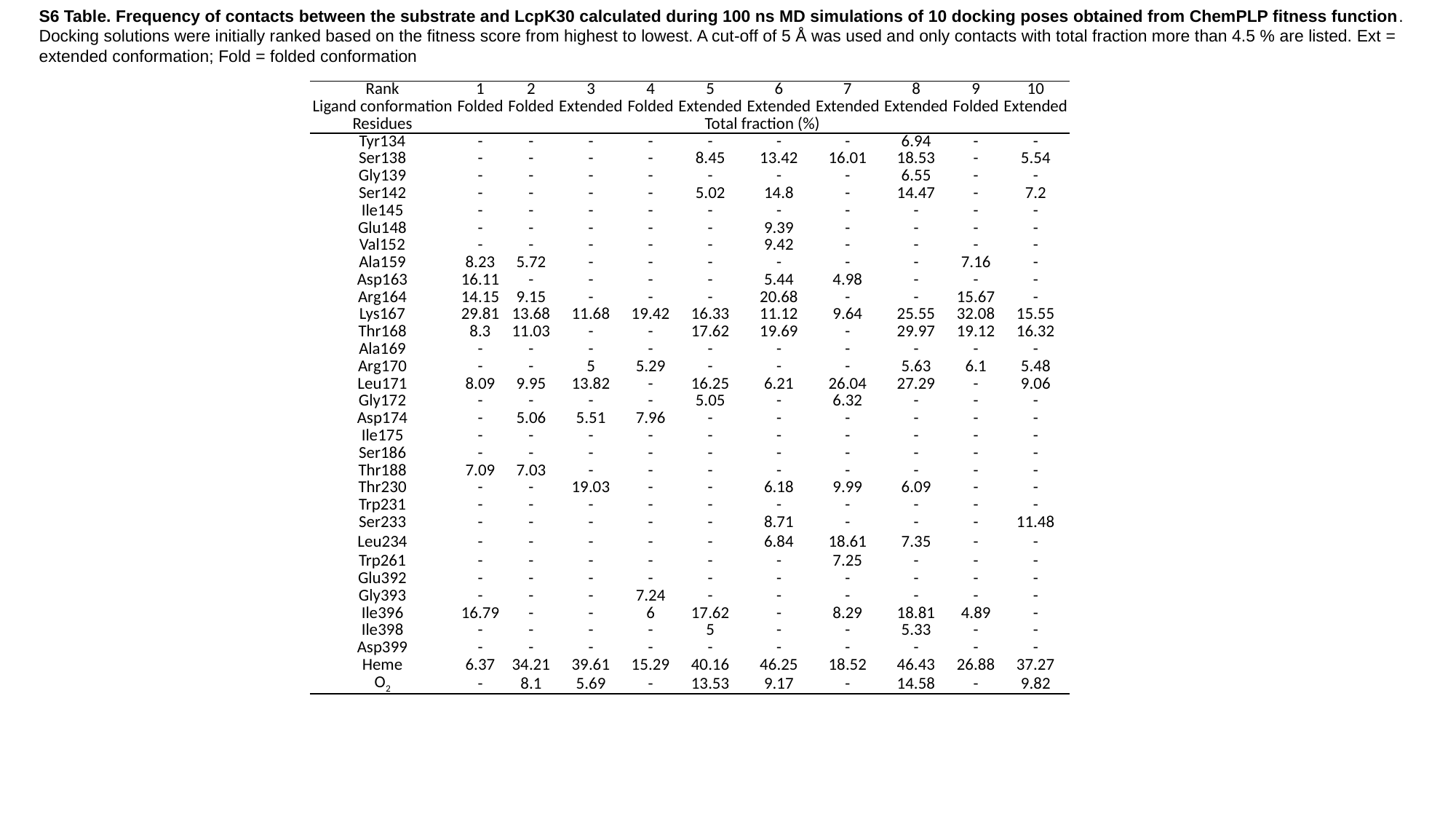

S6 Table. Frequency of contacts between the substrate and LcpK30 calculated during 100 ns MD simulations of 10 docking poses obtained from ChemPLP fitness function. Docking solutions were initially ranked based on the fitness score from highest to lowest. A cut-off of 5 Å was used and only contacts with total fraction more than 4.5 % are listed. Ext = extended conformation; Fold = folded conformation
| Rank | 1 | 2 | 3 | 4 | 5 | 6 | 7 | 8 | 9 | 10 |
| --- | --- | --- | --- | --- | --- | --- | --- | --- | --- | --- |
| Ligand conformation | Folded | Folded | Extended | Folded | Extended | Extended | Extended | Extended | Folded | Extended |
| Residues | Total fraction (%) | | | | | | | | | |
| Tyr134 | - | - | - | - | - | - | - | 6.94 | - | - |
| Ser138 | - | - | - | - | 8.45 | 13.42 | 16.01 | 18.53 | - | 5.54 |
| Gly139 | - | - | - | - | - | - | - | 6.55 | - | - |
| Ser142 | - | - | - | - | 5.02 | 14.8 | - | 14.47 | - | 7.2 |
| Ile145 | - | - | - | - | - | - | - | - | - | - |
| Glu148 | - | - | - | - | - | 9.39 | - | - | - | - |
| Val152 | - | - | - | - | - | 9.42 | - | - | - | - |
| Ala159 | 8.23 | 5.72 | - | - | - | - | - | - | 7.16 | - |
| Asp163 | 16.11 | - | - | - | - | 5.44 | 4.98 | - | - | - |
| Arg164 | 14.15 | 9.15 | - | - | - | 20.68 | - | - | 15.67 | - |
| Lys167 | 29.81 | 13.68 | 11.68 | 19.42 | 16.33 | 11.12 | 9.64 | 25.55 | 32.08 | 15.55 |
| Thr168 | 8.3 | 11.03 | - | - | 17.62 | 19.69 | - | 29.97 | 19.12 | 16.32 |
| Ala169 | - | - | - | - | - | - | - | - | - | - |
| Arg170 | - | - | 5 | 5.29 | - | - | - | 5.63 | 6.1 | 5.48 |
| Leu171 | 8.09 | 9.95 | 13.82 | - | 16.25 | 6.21 | 26.04 | 27.29 | - | 9.06 |
| Gly172 | - | - | - | - | 5.05 | - | 6.32 | - | - | - |
| Asp174 | - | 5.06 | 5.51 | 7.96 | - | - | - | - | - | - |
| Ile175 | - | - | - | - | - | - | - | - | - | - |
| Ser186 | - | - | - | - | - | - | - | - | - | - |
| Thr188 | 7.09 | 7.03 | - | - | - | - | - | - | - | - |
| Thr230 | - | - | 19.03 | - | - | 6.18 | 9.99 | 6.09 | - | - |
| Trp231 | - | - | - | - | - | - | - | - | - | - |
| Ser233 | - | - | - | - | - | 8.71 | - | - | - | 11.48 |
| Leu234 | - | - | - | - | - | 6.84 | 18.61 | 7.35 | - | - |
| Trp261 | - | - | - | - | - | - | 7.25 | - | - | - |
| Glu392 | - | - | - | - | - | - | - | - | - | - |
| Gly393 | - | - | - | 7.24 | - | - | - | - | - | - |
| Ile396 | 16.79 | - | - | 6 | 17.62 | - | 8.29 | 18.81 | 4.89 | - |
| Ile398 | - | - | - | - | 5 | - | - | 5.33 | - | - |
| Asp399 | - | - | - | - | - | - | - | - | - | - |
| Heme | 6.37 | 34.21 | 39.61 | 15.29 | 40.16 | 46.25 | 18.52 | 46.43 | 26.88 | 37.27 |
| O2 | - | 8.1 | 5.69 | - | 13.53 | 9.17 | - | 14.58 | - | 9.82 |
