# Supplementary material for: Computational investigation of cis-1,4-polyisoprene binding to the latex-clearing protein LcpK30
Source: PLoS One. 2024 May 15;19(5):e0302398. doi: 10.1371/journal.pone.0302398 (PMC11095694; doi:10.1371/journal.pone.0302398)
Supplement: Attachment — Submitted filename: Response to reviewers.docx [file pone.0302398.s023.docx]

Dear Editor,

Please find enclosed our revised manuscript ***“Computational investigation of cis-1,4-polyisoprene binding to the latex-clearing protein LcpK30”*** by Aziana Abu Hassan, Marko Hanževački* and Anca Pordea* for consideration for publication as a research article in *PLOS ONE.* Thank you for giving us the opportunity to respond to reviewers comments and to revise our manuscript. We carefully considered and addressed the editor’s and reviewers’ comments and include our response below.

**Journal Requirements**

When submitting your revision, we need you to address these additional requirements.

1. Please ensure that your manuscript meets PLOS ONE's style requirements, including those for file naming. The PLOS ONE style templates can be found at

https://journals.plos.org/plosone/s/file?id=wjVg/PLOSOne_formatting_sample_main_body.pdf and

<https://journals.plos.org/plosone/s/file?id=ba62/PLOSOne_formatting_sample_title_authors_affiliations.pdf>

Response: we used the links above to format our documents and included all supplementary figures and tables as separate files, named and cited appropriately in the text.

2. Please note that funding information should not appear in any section or other areas of your manuscript. We will only publish funding information present in the Funding Statement section of the online submission form. Please remove any funding-related text from the manuscript.

Response: funding text was removed.

3. We note that you have stated that you will provide repository information for your data at acceptance. Should your manuscript be accepted for publication, we will hold it until you provide the relevant accession numbers or DOIs necessary to access your data. If you wish to make changes to your Data Availability statement, please describe these changes in your cover letter and we will update your Data Availability statement to reflect the information you provide.

Response: the relevant accession number for all the data was provided as a link included in the Data Availability Statement at the end of the document. https://doi.org/10.6084/m9.figshare.22941149.

**Reviewers' comments**

We thank the reviewers for their very insightful comments, which allowed us to improve the manuscript. We addressed all comments below. We included additional data and explanations and we moved some of the data from the supplementary information into the main manuscript, as suggested by the reviewers.

**Reviewer #1**

The studied enzymes, latex clearing proteins (Lcps) are interesting candidates for structural studies as they can cleave polymers despite having active catalytic site buried inside the protein structure and furthermore, they can be used to degrade of rubbers and potentially even synthetic polymers. Application of various computational tools, such as CAVER-Pymol plugin 3.0.3, fpocket and Molecular Dynamic (MD) simulations allowed the authors to analyse the structures of Lcps and substrate binding.

**Comment 1.1**

The authors compared the open and closed crystal structures of LcpK30. The open crystal structure of LcpK30 contains, apart from heme, an imidazole molecule in its active site. The open crystal structure in single state is thoroughly analysed by the authors, also considering the probability of acquisition of a polymer in the cavities.

**It is not confirmed, however, if the analysed protein would have the same open conformation during incorporation of a large oligomeric substrate, as it has while incorporating imidazole, which is a smaller aromatic molecule**.

In PET-degrading enzymes that have active site exposed on the surface, it is the binding site’s flexibility that most probably allows the enzyme to incorporate such polymer substrate for depolymerisation. The other example can be found in epoxide hydroxylases, the native long hydrophobic substrate was thought to enter through one tunnel and with proper positioning was reaching exit of the second tunnel. Both tunnels made a L-shape tunnel network. However, during MD study, it was shown, that two domains of the epoxide hydrolase can undergo small conformational changes, and instead of two isolated tunnel, groove can be observed (https://doi.org/10.1016/j.csbj.2021.10.042 ). It is thus possible that LcpK30 undergoes some strong conformational changes to allow easier accommodation of oligomeric substrate to the protein core where the active site is located.

**Such big conformational changes were not possible to be observed by the authors in the performed studies.** The results of the docking studies using GOLD software of flexible model substrate to rigid protein suggest that the protein truly needs to undergo some conformational changes prior to incorporating the substrate, as the obtained poses had very low or even negative fitness score.

Response:

We thank the reviewer for this question because it allowed us to further discuss the importance of the conformational flexibility in LcpK30. To address this question, we carried out extensive 3 x 500 ns MD simulations starting from the closed-like conformation of the enzyme, prepared as described below. In addition to that, to explore conformational changes of the enzyme upon enzyme-substrate complex formation, we performed principal component analysis (PCA) taking into account backbone cartesian coordinates, using MD snapshots from both the open and the closed-like states of the empty enzyme (without substrate bound), as well as of the enzyme in complex with the polyisoprene substrate. New text and data was included in the manuscript, as described below.

Pages 8-10: we introduced changes in title, text and Fig 2 title to clarify differences between static and dynamic models.

Lines 189-196

“We assumed that polyisoprene binding occurs in the catalytically active state of LcpK30 and therefore we prepared an LcpK30 model starting from the open state structure, in which the co-crystalized imidazole molecule was replaced with O_2_ bound to the heme cofactor and where the Lys167 chain (not heme bound) was fully protonated, which we were able to simulate during relatively long simulation timescales. For comparison, we also modelled the closed-like state system, prepared from the closed state structure conformation, but where the coordination of Lys167 to heme was removed and replaced with O_2_ coordination, whilst Lys167 was fully protonated.”

We note that the crystal structure of the closed conformation (PDB 5O1M) most likely resembles the resting state of the enzyme, with neutral Lys167 (instead of O_2_) coordinating heme. We were not interested in simulating this state because the coordination of Lys167 to Fe would prevent binding of the catalytic O_2_ and its subsequent interaction with the substrate.

Lines 198-202

“Relatively low Cα backbone RMSD were obtained over the course of 500 ns MD simulations, for both open and closed-like conformations (see S3a Fig for the open conformation). This suggested that the enzyme with no substrate bound did not undergo large structural changes compared to the reference X-ray structures of the open and closed states.”

Lines 212-244

“Principal component analysis (PCA) taking into account backbone cartesian coordinates was used to compare open and closed states of the enzyme with no substrate. A clear separation of the two states along the PC1 was observed, suggesting that major conformational changes must occur that would allow the transition between closed (positive PC1 values) and open (negative PC1 values) conformations (Fig 3a). Furthermore, the normal mode displacements (Fig 3b) along the PC1 were characterised as conformational changes that predominantly involve helix E residues 160-176, which also contains the central Lys167. Namely, in the open conformation Lys167 is exposed towards the protein surface interacting with the propionates of the heme cofactor, in contrast to the closed-like conformation where it is rotated into the protein interior interacting with O_2_ and Glu148. On the other hand, displacements along the PC2 are associated with the flexibility of the C-terminus loop 392-397 ranging from fully closed (negative PC2 values) to a fully open (positive PC2 values) conformation (Fig 3b).

**Fig 3**. **Principal component analysis (PCA) carried out on structures from MD simulations of open and closed-like states of LcpK30 without substrate**. PCA was performed considering cartesian coordinates of protein backbone atoms (N, Cα, C and O). (a) MD snapshots projected on the first two principal components. Blue and red circles represent MD snapshots belonging to open and closed-like states, respectively. (b) Normal mode displacement vectors associated with the first two principal components showing only motions longer than 2 Å as porcupine in both directions. The backbone is shown as cartoon representation and coloured by the mobility where lower and higher flexibility is depicted in blue and red, respectively.

Despite the closed-like state having identical amino acid protonation states and the same species present in the active site as the open state (fully protonated Lys167 and O_2_ bound to heme), a transition between the two states has not been observed, and the reason for that might be a large barrier separating these two states that simply could not be sampled using conventional MD simulations at the available timescales. This implies that the opening of the enzyme is a complex and controlled process that happens on longer timescales and involves large conformational changes of the helix E and Lys167 and might even be coupled with the approach and the interaction with the polyisoprene substrate.”

We also introduced a principal component analysis to compare the conformations of LcpK30 with and without bound substrate. For this, we combined the MD snapshots for the empty enzyme (no substrate, from analysis above) with MD snapshots of the enzyme in complex with the *cis*-1,4-polyisoprene chain (originating from all simulations of both ChemPLP and ChemScore docking solutions) into a unique PCA plot, now included in S13 Fig. Based on the PCA plots, we concluded the following:

Lines 437-451

“The PCA analysis combining MD snapshots of the enzyme with and without substrate showed that compared to the empty enzyme, the presence of substrate slightly changed the conformation of the enzyme, irrespective of the extended or folded substrate binding mode (S13 Fig). The bound and unbound states were not separated along only one principal component, instead displacements along both PC1 and PC2 were observed. Normal mode displacements along the PC1 included the loop 155-160, helix E 160-176 and C-terminal helix Z2 297-314, whilst the displacement along the PC2 mostly localised at the C-terminal loop residues 392-397. As observed from the RMSD analysis, in some cases where the substrate binds deeper in the active site the conformation of the enzyme bound to substrate was significantly different from the empty enzyme (for example in the case of extended substrate conformations 10 from ChemPLP and 3 from ChemScore). Furthermore, significant structural changes of the enzyme, especially C-terminus loop 392-397, were observed in case of simulations with the folded substrate conformations 1 from both ChemPLP and ChemScore.”

**S13 Fig. Principal component analysis (PCA) carried out on structures from MD simulations of open and closed LcpK30 with and without substrate** PCA was performed considering cartesian coordinates of protein backbone atoms (N, Cα, C and O). (a) MD snapshots projected on the first two principal components. Blue and red circles represent MD snapshots belonging to open and closed-like states, respectively, orange circles represent MD snapshots belonging to the open state LcpK£0 in complex with the substrate. (b) Normal mode displacement vectors associated with the first two principal components showing only motions longer than 2 Å as porcupine in both directions. The backbone is coloured by the mobility where lower and higher flexibility is depicted in blue and red, respectively. Structures from MD simulations projected on the first two principal components considering open LcpK30 (grey) and LcpK30 in complex with substrate from (c) ChemPLP and (d) ChemScore docking poses.

From this data, we conclude that conformational changes occur for the substrate-bound enzyme, compared to either open or closed state without substrate. Simulation of the closed-like state with substrate bound was not possible, because the substrate could not be accommodated within the structure. However, we hypothesise that the binding of the substrate is more likely to occur to the open state than to the closed state, because more space is available within the enzyme (see also answer below, related to tunnels in closed structure).

The reviewer also suggested that there might be a groove forming which would allow easier approach and binding of larger substrates into LcpK30, similar to the one reported for epoxide hydrolase, however we did not observe the formation of such groove from our simulations. We believe that in the case of LcpK30, the tunnel formation is most likely controlled by the conformation of central Lys167 from the helix E and its interaction with the heme propionate groups and by the conformation of the flexible C-terminus loop.

**Comment 1.2**

The authors identified putative tunnels within the crystal structure of LcpK30 only in the OPEN state, using the CAVER-PyMOL plugin. CAVER-PyMOL plugin allows to analyse tunnels in the macromolecule’s core using the geometric approach in single state. The results are strongly influenced by the chosen radius probe. The authors should probably also analyse LcpK30 in the CLOSED state for comparison using CAVER-PyMOL plugin. Moreover, since authors run MD simulations, it could be recommended to use either CAVER implementation for MD simulation analysis or AQUA-DUCT which can more precisely show the potential accessing paths. Authors need to take into consideration, that CAVER and specifically CAVER plugin is using radial probe for tunnels detection and analysis, therefore all asymmetric tunnels cannot be described properly. Instead some of the tunnels has to be merged together. Such problems can be avoided e.g. using water molecules as a small probes – this is especially important for bulky substrates, or substrates which are not globular. Authors can see, what type of analysis is accessible in previously mentioned article https://doi.org/10.1016/j.csbj.2021.10.042 and comparison of geometry based approach (CAVER) with molecules tracking approach (AQUA-DUCT) can be found https://doi.org/10.1021/acs.jcim.2c00985 and https://doi.org/10.1371/journal.pcbi.1010119 In general, the analysis of tunnels in crystal structures only can be misleading. Since authors have MD simulations in hands, the analysis of accessibility to the active site can be done quickly with small effort only.

Response:

No static tunnels were identified using CAVER-Pymol plugin 3.0.3 within the closed state structure (5O1M), using the same calculation parameters as with the open state structure. However, by decreasing the minimum probe radius of tunnel calculations from 0.9 to 0.6 Å, three tunnels were detected, with much lower throughput (0.2 Å) and bottleneck radius (0.6 Å).

We included a clarification at lines 132-133: “We used both the open (5O1L) and the closed state conformations (5O1M), however only the former led to tunnel identification using the default parameters (see Methods).”

We thank the reviewer for suggesting alternative programs for detecting and analysing tunnels from MD simulations. We are aware of the limitations of CAVER software when it comes to characterising wide and asymmetric tunnels, which arise from the fact that the individual tunnels are detected by constructing the Voronoi diagrams using simple spherical probes. However, we strongly believe that accurate tunnel predictions could be obtained by employing the dynamical approach implemented in CAVER Analyst 2 using MD snapshots instead of static crystal structures. In the previously submitted S5a Fig and S2 Table, we have already presented the dynamical tunnel calculations with CAVER Analyst 2 using our MD simulations of the enzyme in open conformation.

We have now moved the analysis of dynamical tunnel clusters for the open state enzyme from the supplementary information into the main manuscript (Table 1 and Fig 4a). In addition to this, we also analysed the features of dynamical tunnel clusters of the closed-like conformation and compared those to the tunnels obtained during MD simulations of open state (Table 1 and S5 Fig). New text and data was included in the manuscript, as described below.

Lines 245-290:

“Given the enzyme flexibility required to accommodate the substrate, we sought to improve the static tunnel predictions by employing the dynamical approach implemented in CAVER Analyst 2, using MD snapshots instead of static crystal structures. The first 5 identified tunnel clusters ranked based on priority (calculated by averaging tunnel throughputs over all snapshots) were analyzed, for MD simulations with both the open and closed-like conformations and the details about the features of the dynamic tunnel clusters are shown in Table 1. Open state tunnel clusters had overall larger average and maximum bottleneck radius, shorter length, smaller curvature and higher throughput than closed state ones, which ultimately makes them more favorable for substrate binding. Furthermore, the priority of the tunnel clusters calculated based on the appearance frequency during the MD simulations was much higher in the open (14-49%) compared to the closed (4-13%) state. Interestingly, when comparing the spatial distribution of timeless tunnel cluster pathways, we could clearly see how the central Lys167 plays an important role in separating tunnels into two wider superclusters in the open state (Fig 4a, clusters 1 and 3 forming the green supercluster and clusters 2 and 5 forming the blue super-cluster). This is in contrast with the rotated Lys167 conformation forcing the appearance of one narrow supercluster in the closed-like state (S5 Fig, clusters 1,3,4 and 5 forming the limon supercluster).”

**Fig 4. Dynamical tunnel mapping and pocket identification in the LcpK30 open structure without substrate.** (a) Dynamical tunnel calculations with CAVER Analyst 2 using MD simulations of LcpK30 in open conformation. First 5 dominant tunnel clusters were calculated on 1500 snapshots from MD simulations of the open state. Two superclusters are formed, colored in green (comprised of clusters 1 and 3) and blue (comprised of clusters 2 and 5). Clustering displays the center lines for all tunnels computed for all snapshots at once. Centre lines are colored according to their related clusters. (b) Pockets detected closest to heme calculated on 1500 snapshots from MD simulations of LcpK30 in the open state.

**Table 1**. **Features of first 5 dominant dynamical tunnel clusters in LcpK30 open and closed-like states**. Calculations were performed on 1500 snapshots from MD simulations of open (upper) and closed-like (lower) states of LcpK30.

We note that we changed the tunnel cluster notation from the initially submitted manuscript, where we referred to the green supercluster as cluster 1; to the blue supercluster as cluster 2; and to the narrow cluster 4 as cluster 3.

To investigate the effects of the bound substrate on the tunnels leading to the active site of the enzyme we introduced a new Table 3 and the corresponding Figure 6, in which we presented the results from the CAVER Analyst 2 calculations of dynamical tunnel clusters using snapshots from MD simulations of the complex between LcpK30 and *cis*-1,4-polyisoprene substrate in extended and folded conformation originating from both ChemPLP and ChemScore poses. We concluded the following:

Lines 459-474:

“The effects of the bound substrate on the tunnels leading to the active site were investigated next, by calculating the dynamical tunnel clusters within the complex between LcpK30 and cis-1,4-polyisoprene in extended and folded conformations, originating from both ChemPLP and ChemScore poses. The calculated features of the first 10 dominant clusters demonstrated that although throughputs were relatively high, and the priority of the individual tunnel clusters was very broad ranging from 9-44%, these tunnels had overall similar average and maximum bottleneck radius as in the case of LcpK30 in the closed-like state and without substrate bound (Fig 6 and Table 3, compare with Fig 4 and Table 1). However, the spatial distribution of tunnel clusters was similar to the open conformation without substrate, with two clearly separated superclusters due to the presence of the salt bridge between propionates of the heme cofactor and Lys167. All these results indicated that although mutual conformational changes of the enzyme and the substrate occurred (as supported by the PCA analysis) which influenced the through-put of the tunnels, a bound polyisoprene substrate strongly interacted with the enzyme within the characterised tunnels, which caused the narrowing of the bottlenecks.”

**Table 3**. **Features of first 10 dominant tunnel clusters in LcpK30 bound to substrate**. Calculations were performed on 1000 snapshots from MD simulations of open state LcpK30 with *cis*-1,4-polyisoprene substrate bound. The tunnels were calculated starting from central Fe atom of heme and excluding O_2_ and substrate.

**Fig 6.** **Dynamical tunnel mapping and pocket identification in the LcpK30 open structure with bound substrate.** First 10 dominant tunnel clusters were calculated on 1000 snapshots from MD simulations of the open state LcpK30 with *cis*-1,4-polyisoprene substrate bound, using CAVER Analyst 2.

Because we believe these results are important and demonstrate why open conformation might be more suitable for the substrate binding and how the substrate binding influences the tunnels features in LcpK30 we decided to include this comparison in the main manuscript.

**Comment 1.3**

Plot in fig.3 a should not be a line plot, but a point plot.

Response

We modified the line plot in the Figure into a bar plot, which we felt offered a better visualisation than a point plot. The figure is now Fig 8.

Fig 8. Residues that frequently interact with the substrate during MD simulations. (a) Close contacts between frequently occurring bound conformations of the substrate (extended and folded) and LcpK30, calculated from 100 ns MD simulations of 20 docking poses obtained with ChemPLP and ChemScore fitness function. The profile was constructed by averaging total fractions obtained for similar conformations. Conformations were clustered based on the contacts and the features from the visual inspection; (b) Representative snapshots extracted from MD simulations showing the main extended (ChemPLP pose 10 and ChemScore pose 5 shown as dark green and light green licorice, respectively) and folded (ChemScore pose 1 shown as magenta licorice) conformations and protein residues (shown as lines) that interact with the *cis*-1,4-polyisoprene substrate model bound to LcpK30 near heme (sphere representation). The three tunnels and the hydrophobic cavity are shown as blue, green, orange and yellow surface, respectively. The rest of the LcpK30 and hydrogen atoms are omitted for clarity.

**Comment 1.4**

In lines 504-507 and 567-570 the authors mention experimental results of distribution of oligomers produced by LcpK30. It is not clear however, what results they mean exactly (citation?) and what are the oligomers produced by LcpK30 and which part of the modelled substrate they constitute.

Response

We refer to experimental data that was previously reported by others, in which oligomers produced by LcpK30 were isolated and quantified by LC-MS and HPLC. We have now included the following clarifications:

Lines 81-83 in the introduction, “with a preference for 5-6 (for Lcp1VH2) [14] or 5-8 (for LcpK30) [1,15,16] isoprene repetitive units” was changed to “with a preference for 5-6 (for Lcp1VH2) or 4-7 (for LcpK30) isoprene repetitive units and lower concentrations of smaller and larger products”.

Lines 588-594:

“Fig 10 shows that the central C=C double bonds (numbered 5 and 6) of the substrate were more frequently found in the near vicinity of heme-O_2_ and therefore more prone to oxidative cleavage into two oligomers of 4 and 5 isoprene units. This can partially explain the previously reported experimentally observed distribution of oligomers produced by LcpK30 which shows a preference for products containing 4-7 intact isoprene units [1,15,16].

Lines 638-640:

“Furthermore, an oxidative cleavage frequency profile was constructed, which can partially explain previously reported experimental results showing cleavage into mainly 4-7 intact isoprene units.”

Lines 653-658:

“A second binding mode with the substrate in a folded conformation also occurred, with a central double bond in proximity to the heme-bound oxygen, leading to a preference for oligomers with 5 to 6 C=C bonds, which can partially explain previously reported experimental results showing cleavage into mainly 4-7 intact isoprene units.”

**Comment 1.5**

In lines 589-592 of the methods section, the authors mention that they remove water molecules from the crystal structure of LcpK30. Then in lines 677-680 they describe the solvation of protein with OPC water using the truncated octahedron box. **Prior to solvation, the authors should add the crystal water as to ensure the correct water networks inside the protein core. In the case of holo system, only crystallographic waters producing clashes with the docked substrate, should have been removed.**

Such procedure is much safer, and the equilibration time is shorter. In case when whole buried water is removed from the interior, some cavities might initially be closed, and longer time is required to expand them to size accommodating water again. Authors can also used 3D-RISM and Placevent for system preparation. More details can be found in papers dedicated to mentioned methods or in review describing methods using water molecules in computational analysis (<https://doi>.org/10.1016/j.csbj.2020.02.001).

Response

We want to thank the reviewer for this comment, and we agree that water network analysis in proteins is important and could provide useful insights into stability of the system. We are aware that different practices and tools are used when solvating and preparing the proteins for MD simulations, and generally it is wise to keep buried functional crystal water molecules that may play an important role in structural stabilization or catalytic function. In our case we are confident that keeping crystal water molecules would not significantly change the outcome of our simulations as the majority of the co-crystalised waters reported in the LcpK30 crystal structure are close to or at the surface of the enzyme and are easily accessible, therefore easily exchanged with the rest of the bulk.

Typically, the absence of structurally important water molecules in MD simulation might cause collapse of artificially created vacuum pockets in the protein interior and aggregation of the protein backbone and/or sidechains accompanied with a large structural changes of the protein from its reference X-ray structure. Some of the common analyses of the protein stability during MD simulations such as root mean square deviation (RMSD) and root mean square fluctuation (RMSF) indicate how much protein structure changes from a reference structure and which residues are involved in these fluctuations. For example, the larger the RMSD values the larger the structural changes from the reference point. Here we observed overall low protein Cα backbone RMSD compared to the initial crystal structure indicating that the enzyme did not change significantly compared to the X-ray structure, as shown in S3a Fig.

However, because little is known about the importance of water molecules for the substrate binding and catalysis in LcpK30, we carried out additional analysis that could tell us whether more buried water molecules present in the crystal structure are recovered in our simulations. Shortly after the relaxation, several buried protein sites, including the active site, were solvated, which was confirmed by obtaining a similar water occupancy from MD simulations as in the crystal structure. Especially interesting were two buried crystal water molecules participating in the hydrogen bonding network with the delta-proton at the proximal histidine attached to the heme cofactor. Despite these particular water molecules not being present at the beginning of the simulation, we observed their approach to the same histidine site shortly after the initial unrestrained NPT relaxation. Therefore, buried sites such as the one near the proximal histidine in LcpK30 strongly favour the presence of water molecules.

To further demonstrate why the open conformation might be more suitable for the substrate binding we investigated the probability of water molecules approaching into the hydrophobic active site of LcpK30 by calculating the radial distribution function (RDF) of water oxygen atoms around the heme-bound O_2_, and we included this in S6 Fig. From the RDFs we could conclude that the approach of water molecules into the active site of LcpK30 is more feasible in the open conformation, which could also suggest easier binding of larger substrates into the open compared to the closed conformation. Moreover, we also observed increased hydration of the previously characterised tunnels that connect the active site with the bulk in the open state compared to poorly hydrated tunnels in the closed state which again suggests that these tunnels are more feasible in the open structure, which could also suggest easier binding of larger substrates into the open compared to the closed conformation. Namely, due to predominantly hydrophobic interaction between a bound substrate and the active site, the highly energetic polar water molecules initially found occupying the protein interior should be easily replaced by the lipophilic substrate (dehydration of the active site strongly favours complex formation which is demonstrated by favourable binding energy calculations presented in Fig 9).

To address the comment, we included the following text at lines 297-312, supported by the newly introduced S6 Fig.

“Interestingly, despite removing crystal waters from the crystal structure before the simulation, we observed a water network occupying the tunnels and both hydrophobic pockets above heme. Shortly after the relaxation, several buried protein sites, including the active site, were solvated, which was confirmed by obtaining a similar water occupancy from MD simulations as in the crystal structure. Moreover, we observed increased hydration of the tunnels in the open state compared to poorly hydrated tunnels in the closed state, again suggesting that these tunnels are more feasible in the open structure. This was also supported by the radial distribution function (RDF) of water oxygen atoms around the heme-bound O2 indicating that the probability of water molecules approaching into the hydrophobic active site of LcpK30 is higher in the open conformation (S6 Fig).

Taken together, these results suggested an easier binding of larger substrates into the open compared to the closed conformation and indicated the existence of hydrophobic tunnels that provide access of the polymeric substrate to the active site. Next, we used docking of a polyisoprene substrate model to the open state structure to gain an understanding of the binding mode of the substrate to LcpK30.”

**S6 Fig**. **Dynamic of water molecules during simulations of open and closed-like states of LcpK30**. Radial distribution function (RDF) of water oxygen atoms from O_2_ molecule bound to heme in (a) open and (b) closed LcpK30. The isosurface (isovalue = 100) showing the density of water oxygen atoms around the active site in (c) open and (d) closed LcpK30. Top view at the heme cofactor in the active site of LcpK30 showing water molecules as blue surface using the representative MD snapshots in (e) open and (f) closed LcpK30. The analysis was carried out on 6000 frames from 3 repeat MD simulations of open and closed-like LcpK30 states.

**Overall comments**

Overall, the study gives interesting insight into LcpK30 structure, tunnel and pocket network and their dynamics, and provides hypotheses on how Lcps can incorporate and cleave oligomeric substrates. The authors identified probable tunnels used for transport of the oligomeric substrates to the active site. However, there are doubts if analysis was done deep enough to provide true description of the substrate accessibility.

Article provide important findings, as the tunnel-lining residues are suitable targets for mutagenesis aimed at improving enzymatic degradation rates. The information on tunnels structure and dynamics mostly comes from analysis of open structure of LcpK30. However, the MD simulations should provide insight into much more opened structure. Authors has mentioned helix E as important one – on the second side of the heme, we can see C-terminal loop, which is stabilised by short helix (71-81). It is quite probable, that this sandwich can open more significantly. The second tunnel will remain separated by the loop. Authors has identified I396 as a one of the interacting residues – it is the one which separates two distinctive tunnels from CAVER analysis.

**The authors could compare the closed structure as well and tunnels dynamics in the structures with bound substrate to more deeply understand what conformational changes the LcpK30 enzyme undergoes and how incorporation of a big oligomeric substrate influences tunnel dynamics.** This was partly done by RMSD and RMSF analysis, but **tunnel radius investigation in a enzyme-substrate complex could give insight into tunnel bottlenecks and residues that might interfere in the substrate/product transport**.

Again, we thank the reviewer for the insightful comments and suggestions. We included additional simulations and analyses with the enzyme in both the open and the closed state, and we also compared the empty enzyme with the enzyme bound to substrate, in an attempt to clarify the changes in conformation occurring upon substrate binding. These results are summarised above.

**Reviewer #2**

The manuscript by Hassan and co-workers brings an interesting approach to the investigation of the polyisoprene binding to latex-clearing proteins and indeed might represent a good contribution for a better understanding of the mechanisms behind the action of this type of protein. The submitted version of the manuscript, however, seems to show some flaws that don't permit ensuring publication. Some suggestions of improvements are listed below:

**Comment 2.1**

The english language is good but small typos and grammar issues were detected. I recommend a deep revision before resubmission;

Response: Thank you for the suggestion. We checked the manuscript and revised typos and grammar issues.

**Comment 2.2**

I don't see need of an author summary. Unless this is a request from the journal I suggest deleting it;

Response: We removed the author summary.

**Comment 2.3**

I suggest no repeating colors in Figs. 2a and 2b. This can confuse the readers. Use different colors for tunnels and pockets;

Response: We modified colors of pockets 1 and 3 in Fig 2b from green and blue to olive and cyan, respectively.

**Fig 2. Static tunnel mapping and pocket identification in the LcpK30 open structure**. (a) Two dominant tunnels identified by CAVER-Pymol plugin 3.0.3 (tunnel 1 in blue and tunnel 2 in green); (b) five detected pockets or cavities closest to heme in the LcpK30 static X-ray structure identified by automated geometry-based fpocket tool. The pockets are pocket 1 (olive), pocket 3 (cyan), pocket 4 (orange), pocket 9 (magenta) and pocket 12 (yellow).

**Comment 2.4**

The docking protocol is poor. There is no re-docking to validate the docking protocol neither an appropriate description about the number of runs, criteria to select the best poses, algorithm used, etc.... Also there is no experimental support for the binding of the ligand. Authors should use some experimental evidence that justify the poses chosen. If it's not possible, this should be clarified in the text and the speculative character of the work evidenced.

Response:

The docking protocol used in this study used the default parameters in GOLD (see also refs 25 and 35). Rather than re-docking, we used all 10 poses obtained with the ChemPLP and Chemscore scoring functions as initial binding conformations for the MD simulations. This approach gave us reassurance that we did not bias the selection of relevant poses.

The default parameters and the algorithm for the docking are included in the Supplementary Files (GOLD-DOCKING). In each folder of docking runs there is a Notepad file entitled gold_protein which lists all the parameters used during the docking run. The below table shows the docking attempts that were performed before selecting the poses for MD simulations. The scoring functions were chosen after carefully comparing and examining the poses based on the distance between the C=C bond and the distal oxygen atom of the Fe-O_2_ complex. The docking solutions obtained with ChemPLP and Chemscore scoring functions mostly gave a distance of less than and near to 5 Å, which gave an indication of potentially catalytically relevant poses. This was included in lines 363-366 and lines 720-723 in the manuscript.

| **Scoring Function** | **Type of docking** | **Defined binding site (Å)** |
| --- | --- | --- |
| chemplp/default | Rigid | 10 and 15 |
|  | Flexible | 10 and 15 |
| Chemscore/default | Rigid | 10 and 15 |
|  | Flexible | 10 and 15 |
| Chemscore/p450_pdb | Rigid | 10 |
|  | Flexible | 10 |
| Goldscore/default | Rigid | 10 |
|  | Flexible | 10 |
| Goldscore/p450_pdb | Rigid | 10 |
|  | Flexible | 10 |

Experimental evidence on ligand binding is not available in the literature and therefore we cannot compare our predictions against experimental data. We included the following clarifications to evidence the speculative nature of the work:

Lines 332-334:

“In an initial approach, we used docking to get an approximate indication of the binding of this substrate model to LcpK30, which we further refined with molecular dynamics simulations (see next section).”

Lines 667-671:

“Whilst experimental evidence is required to confirm the substrate binding modes, the results presented here provide valuable hypotheses on how latex clearing proteins can incorporate and cleave oligomeric substrates and suggest suitable targets for mutagenesis aimed at improving enzymatic degradation rates.”

**Comment 2.5**

How many poses were selected for the MD simulations? this is not clear in the manuscript. Why to select folded and extended poses? There is no hint in the literature pointing to one or another? This should be discussed in the text;

Response:

Thank you for this comment, which allowed us to make clarifications and improve the readability of the manuscript. We carried out simulations for all top 10 ranked solutions obtained with each of the ChemPLP and ChemScore fitness function (20 poses in total). This information was briefly included at the beginning of the MD simulations section, and we have now slightly expanded it. For clarity, we now also included this information in the Methods section.

We did not select extended and folded conformations. We inspected all the 20 docking poses of the isoprene decamer, which showed different conformations. We grouped these into two main groups of similar conformations, labelled as extended and folded due to their appearance inside the protein. We then performed MD simulations using all of these initial poses, to assess which conformations are likely to be more viable. It is not uncommon for polymers to adopt several conformations when docked into enzymes (see the case for PET here: <https://doi.org/10.1080/07391102.2023.2240893>).

**Comment 2.6**

Most docking programs state favorable docking scores as negative energy values. If GOLD is different this should be clarified in the text;

Response

We included additional clarifications in the text at the beginning of the docking section, lines 337-344

“These scoring functions calculate docking scores by employing different empirical energy terms, such as van der Waals (VDW) energy, electrostatics, hydrogen bond, desolvation, entropy and hydrophobicity, to yield a dimensionless fitness score, which is optimised for ligand binding site prediction over binding affinity prediction. Hence, the fitness score provides an indication of the quality of the docking position. A higher (positive) fitness score indicates a higher probability that a ligand will bind to a protein in the given docking position, whilst a negative score indicates poor binding [25].”

**Comment 2.7**

Many important results are shown as supplementary material. This makes it hard to follow the discussion. **I recommend revising it and bringing back to the text the most relevant figures and tables.**

Response

Thank you for this suggestion, which allows us to improve the readability of the manuscript. We have moved the following information from supplementary into the main manuscript.

Table 1. Features of dynamical tunnel clusters, which has now been updated to include tunnels of the closed-like state.

Fig 4a. Dynamical tunnel mapping in LcpK30 open state.

Fig 5. RMSD and RMSF profiles for LcpK30 in the open state with bound substrate.

Fig 7. Superimposed snapshots from MD simulations of substrate.

Fig 9. Linear interaction energy analysis.

**Comment 2.8**

Most of software and methods used are not properly cited in the manuscript;

Response

Response

We have now checked that all software and methods are properly cited, and we included all relevant references in the Methods section. However if this is still a problem we welcome suggestions for revisions. In particular, we have referenced:

Line 680: Caver-Pymol plugin

Line 690: Caver Analyst 2

Line 694: fpocket

Line 704: GOLD

Line 727: PLIP

Lines 728-789: MD methods and programmes

We checked that all software was properly cited, however if this is still a problem we welcome suggestions for revisions.

**Comment 2.9**

Author mention binding energy calculations during the MD simulations but don't present any result.

Response

Results were presented in the SI (former Figure S16) and have now been included in the main manuscript as Fig 9.

Thank you in advance for your consideration of our manuscript.

Yours sincerely,

Anca Pordea
